# Label-free linear and non-linear vibrational spectroscopy for functional materials: state-of-the-art and future perspectives

**DOI:** 10.1039/d5sc04114g

**Published:** 2025-11-04

**Authors:** Michael Freduah Agyemang, Akuila L. J. L. Edwards, Stefan Zechel, Martin D. Hager, Michael Schmitt, Juergen Popp

**Affiliations:** a Institute of Physical Chemistry (IPC), Abbe Center of Photonics (ACP), Friedrich Schiller University Jena Helmholzweg 4 07743 Jena Germany juergen.popp@uni-jena.de; b Leibniz Institute of Photonic Technology e.V. Jena, Albert-Einstein-Str. 9 07745 Jena Germany; c Laboratory of Organic and Macromolecular Chemistry (IOMC), Friedrich Schiller University Jena Humboldstr. 10 07743 Jena Germany; d Jena Center of Soft Matter (JCSM), Friedrich Schiller University Jena Philosophenweg 7 07743 Jena Germany; e HIPOLE Jena Philosphenweg 12-14 07743 Jena Germany; f Helmholtz Zentrum Berlin für Materialien und Energie GmbH Hahn-Meitner-Platz 1 14109 Berlin Germany

## Abstract

Functional materials, with their specific properties and functions, are instrumental in advancing technological applications across diverse fields such as biomedicine, energy, aerospace, and electronics. To drive innovation in these materials, it is essential to understand their molecular and structural compositions and to correlate these to their switchable macroscopic properties. This correlation is fundamental to their development and optimization. In this context, label-free linear and non-linear vibrational spectroscopy techniques such as infrared (IR) absorption and Raman spectroscopy are invaluable. These techniques offer molecular-level insights into the composition, structure, and dynamic molecular behavior of these functional materials. Moreover, they enable non-invasive, detailed analyses, which are critical for preserving the integrity of these materials during study. This article explores how state-of-the-art label-free linear and non-linear vibrational spectroscopy has effectively been applied in the study of functional materials. It highlights the necessity of these techniques and discusses their many advantages. Notably, their ability to deliver real-time, high-resolution, and non-destructive insights into the molecular and functional properties of functional materials makes them indispensable techniques. Consequently, these methods are accelerating innovation and paving the way for breakthroughs in the design and application of functional materials.

## Abstract 2

Vibrational spectroscopy combined with various analytical techniques like 2Dcos, DFT, AI, and machine learning – powerful and versatile technique applicable to all sample types.

## Graphical abstract

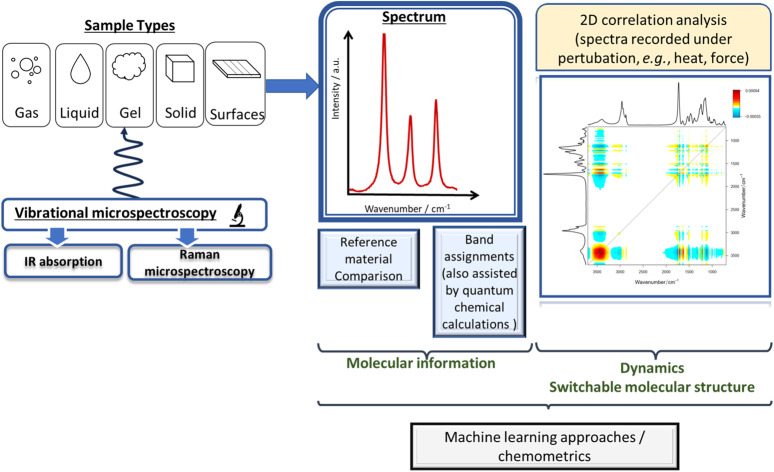

## Introduction

In recent years, technological and industrial applications have experienced transformative advancements largely due to functional materials. Unlike conventional materials, which are mainly designed for their mechanical integrity and stability and often lack the adaptability and responsiveness needed for modern applications, functional materials overcome limitations such as inflexibility, inability to self-heal, and lack of responsiveness to external stimuli.^1,2^ Functional materials feature specific properties (*e.g.*, electrical, optical properties). Due to these properties these materials can have specific functions, which are beyond structural properties. There are several functions like energy storage or conversion, sensing and actuation. Some of these functions require stimuli-responsiveness, *i.e.* shape, color, stiffness, or thermal conductivity can change in response to external stimuli such as stress, temperature, moisture, pH, electric, or magnetic fields, which highlights their adaptiveness.^1–3^ An example of this are piezoelectric materials, which generate an electric current when mechanically stressed^4–6^ or an actuator that can create torque or displacement under the influence of an electrical voltage.^7–10^ Furthermore, photochromic materials that can change their optical properties when exposed to light can be mentioned in this context.^11–13^ Their applications span from aerospace engineering, where they contribute to adaptive structures,^4,14^ biomedical devices, where they enable targeted drug delivery systems,^15–17^ and to the energy sector, where they enhance the efficiency and functionality of renewable energy conversion and storage system.^18–21^

From a sustainability standpoint, functional materials offer significant advantages by reducing waste, improving energy efficiency, and extending the lifespan of products.^22–25^ Shape-memory polymers, for instance, can revert to their original shape after deformation,^22,23,26^ reducing the need for replacements, while self-healing materials autonomously repair damages,^24,25,27^ thereby minimizing maintenance and resource consumption.

This underscores the importance of characterizing and analyzing these functional materials for the advancement of sustainable technologies and infrastructure across various sectors. Vibrational spectroscopy—both linear and non-linear—plays a key role in this context, as it enables label-free analysis, allowing materials to be examined without alteration or damage. Linear vibrational spectroscopy, such as infrared (IR) absorption and spontaneous Raman spectroscopy inclusive Raman signal enhancing methods (*e.g.*, surface-enhanced Raman (SERS) or resonance Raman spectroscopy), provides detailed information about molecular vibrations and material composition which are essential for understanding the fundamental properties and behaviors of these materials.^28–30^ Building on these linear methods, advances in ultrafast laser technology and nonlinear optics have enabled the development of nonlinear vibrational spectroscopic methods that access higher-order light–matter interactions and multidimensional information. In the context of IR spectroscopy, sum-frequency generation (SFG)^31–33^ and two-dimensional infrared spectroscopy (2D-IR)^34–36^ are particularly noteworthy, as they provide complementary insights into interfacial order, site-specific coupling, and ultrafast structural dynamics. SFG isolates non-centrosymmetric interfaces (*e.g.*, solid/liquid, material/air) whereas 2D-IR disentangles congested bands and reports on energy flow and structural dynamics.^31,35,36^ In the Raman domain, coherent Raman techniques—most prominently coherent anti-Stokes Raman scattering (CARS) and stimulated Raman scattering (SRS)—extend vibrational spectroscopy toward label-free imaging with high spatial and temporal resolution, enabling the investigation of dynamic processes and complex interactions within functional materials.^28,29^ Overall, vibrational spectroscopy, thus, provides a comprehensive framework to probe composition, structure, and dynamics across multiple length and time scales, facilitating a deeper understanding of material functionality and potential applications.

In this review, we will explore the state-of-the-art advancements in label-free linear and non-linear vibrational spectroscopy as applied to functional materials. We will discuss the necessity and advantages of functional materials, their production technologies, and the latest developments in their applications. Finally, we will delve into how linear and non-linear vibrational spectroscopy techniques contribute to the understanding and innovation of functional materials, paving the way for future breakthroughs in this exciting field.

## Vibrational spectroscopy and analysis

**Fig. 1:**
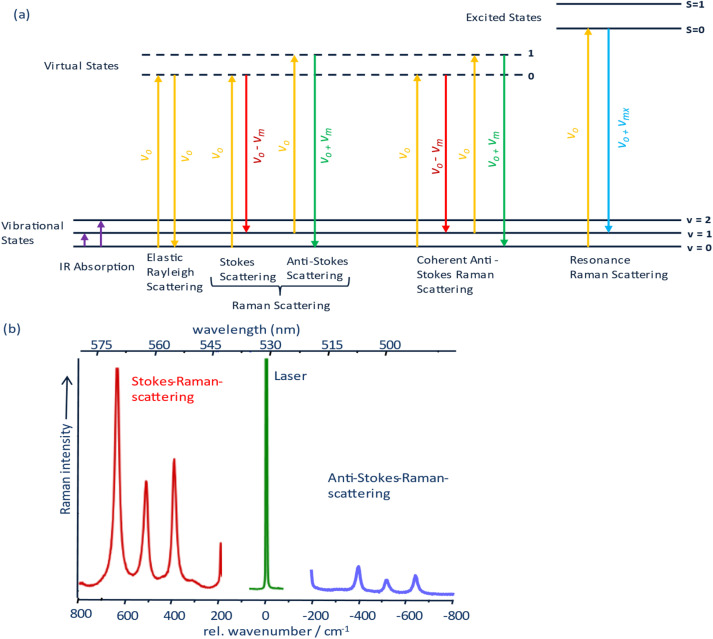
(a) Jablonski diagram showing the transitions from IR absorption, Rayleigh elastic scattering, spontaneous Raman scattering, coherent anti-Stokes Raman scattering and resonance Raman scattering where *v*_o_ are the frequency of the incident photon (laser light), *v*_m_ is the frequency of the molecular vibration and *v*_mx_ is the molecular vibration that is resonantly enhanced. (b) Spectra showing Stokes, anti-Stokes, and Rayleigh scattering from titanium dioxide (TiO_2_). Note that the intensity of the Rayleigh scattering peak has been truncated for better visualization.

Spectroscopy encompasses a broad set of techniques that analyze the interaction between electromagnetic (EM) radiation and matter.^37,38^ These interactions enable the observation of transitions between quantized energy states in atoms and molecules – ranging from rotational and vibrational to electronic levels (as illustrated in Fig. 1) – thereby providing a molecular fingerprint of the analyte.^37,39–41^ Vibrational spectroscopy, in particular, probes molecular vibrations associated with chemical bonds, enabling non-invasive, label-free identification of molecular structures and dynamics.^40,42^

A molecule consisting of N atoms possesses 3N degrees of freedom, representing motion in three-dimensional space. These are partitioned into translational, rotational, and vibrational modes. For nonlinear molecules, 3N-6 degrees of freedom describe internal vibrational modes, referred to as “normal modes”, which are fundamental to vibrational spectroscopy. These vibrational modes are characteristics of the chemical bonds and geometrical structure of the molecule, forming a unique spectral fingerprint that can be exploited for material identification and characterization.^40–42^

Quantum mechanically, molecular vibrations are often approximated by harmonic oscillators, wherein energy levels are quantized and equidistant. However, real molecular systems deviate from this ideal, particularly at higher vibrational levels, and are better represented by anharmonic oscillators. This anharmonicity leads to phenomena such as overtones and combination bands – features that provide additional structural information but also complicates spectral interpretation. The effective mass and force constant associated with a given vibrational mode determines its energy and spectral position.^43–45^

Normal modes are further classified into stretching (valence) and deformation (bending) vibrations. Stretching vibrations typically involve changes in bond lengths and occur at higher wavenumbers (>1500 cm^−1^), while bending vibrations involve bond angle variations and appear at lower wavenumbers. These motions can occur symmetrically or asymmetrically, in or out of molecular planes, and their specific symmetry properties determine their activity in infrared (IR) absorption or Raman scattering.

Molecular vibrations can be excited *via* two principle mechanisms: infrared absorption and Raman scattering. In IR spectroscopy, vibrational transitions are induced by the direct absorption of mid-infrared light (Fig. 1). For a vibration to be IR-active, it must result in a change in the molecule's dipole moment during the vibrational motion. This makes IR spectroscopy particularly sensitive to polar bonds and functional groups. However, one limitation is its sensitivity to water. Due to water's strong IR absorption, its presence can dominate the spectrum and obscure signals from other components in the system.^42,45^

Fourier-transform infrared (FTIR) spectroscopy is the most widely used IR technique. It uses an interferometer to record the spectral response of the sample over a broad range of frequencies simultaneously, improving signal-to-noise ratio and reducing acquisition time. FTIR provides high-resolution spectral data and is suitable for various sample forms (solid, liquid, or gas).^46–48^

Attenuated Total Internal Reflection (ATR) is a powerful extension of FTIR. In ATR-FTIR spectroscopy, IR light is internally reflected within a highly-refractive-index crystal, creating an evanescent wave that penetrates only a few micrometers into the sample. This makes ATR especially advantageous for analyzing thin films, coatings, or surface layers without extensive sample preparation. Moreover, it allows for direct measurement of solid and liquid samples with minimal interference from sample thickness or homogeneity.^48^

Conventional IR spectroscopy methods can be coupled to a microscope for chemical imaging, but in the far field the spatial resolution is fundamentally limited by diffraction (the Abbe limit) and so even at best, one can resolve only a few micrometers.^49–51^ These setups also struggle with thick or opaque samples and with glass substrates, leaving, *e.g.*, fine material microdomains to remain blurred (coarse spatial resolution).^52^ To overcome these physical and practical limits, sub-Abbe-IR approaches such as optical photothermal infrared spectroscopy (OPTIR),^52,53^ atomic force microscopy-infrared (AFM-IR),^54,55^ photo-induced force microscopy (PiFM),^56,57^ and scattering-type scanning near-field optical microscopy (s-SNOM)^50,58^ are applied.

OPTIR is a pump-probe technique for both spectroscopy and imaging.^52,53^ A pulsed, tunable mid-IR pump laser is used to excite specific molecular vibrations causing absorption-induced heating resulting in a thermal expansion and in turn local change in refractive index as a consequence of optical energy transfer to the sample while a second co-aligned visible probe laser (usually 532 nm), detects these photothermal changes yielding an IR absorption spectrum with sub-micrometer spatial resolution—well beyond the diffraction limit of conventional IR microscopy.^52,59,60^

AFM-IR, PiFM, and s-SNOM are nano-IR spectroscopy techniques that extend from the conventional AFM that provide nanoscale chemical specificity, which conventional AFM^61^ lacks. The key idea is that the AFM tip not only provides topographic information but also acts as a nanoscale optical antenna or probe the localized light–matter interactions well below the diffraction limit since the radius of the tip is the only factor that limits spatial resolution.^54^

In AFM-IR, a pulsed tunable mid-IR laser which is tuned to match the absorbing band of the sample, heating the sample locally, causing a rapid photothermal nanoscale expansion due to the sample absorption, driving the AFM cantilever (typically in contact mode) to oscillate in amplitude.^54,55^ A local IR spectrum is generated by monitoring the amplitude of the cantilever producing a signal proportional to the IR absorption. AFM-IR can map IR-induced thermal expansion with about 20 nm spatial resolution by scanning the AFM probe across the surface and monitoring the cantilever amplitude at a fixed wavenumber thereby yielding chemically specific images.^59,62^ The trade-offs with AFM-IR are speed and sample mechanics as a single high-quality frame often takes tens of minutes to acquire and the method relies sufficiently on absorption and stable tip and thus very rough or ultrasoft surfaces or liquid environments can be challenging.^59^ This is where PiFM becomes relevant. PiFM operates in tapping mode and sensing light-induced polarization forces rather than thermal expansion. The metal-coated AFM tip creates a highly local enhanced field from the excitation laser and then locally polarizes the sample, resulting in tip sample electromagnetic forces that are detected by oscillation of the cantilever using a feedback laser that are detected with a quadrant photodiodePiFM.^56,57^ PiFM obtains high spatial resolution in the nanoscale of about 10 nm chemical map and AFM topography concurrently with excellent signal to noise ratio and small interaction volume.^56^

In s-SNOM, the AFM tip is used as a nanoantenna, which when illuminated with a tunable or broadband IR, the near-field scattered light is detected interferometrically (often pseudoheterodyne) to retrieve amplitude and phase properties of the scattered light as a function of the refractive index.^50,58^ By scanning across the sample while detecting the elastically scattered light, optical response of the sample is imaged. The local light–matter interaction under the AFM tip provides the electric field confinement and enhancement to achieve sub- 10 nm spatial resolution.^50,58^

Together, these nano-IR techniques push IR imaging well beyond the diffraction limit.

Raman spectroscopy, by contrast, relies on inelastic scattering of monochromatic light.^63^ When a photon interacts with a molecule, most scatter elastically (Reyleigh scattering), but a small fraction interacts with molecular vibrational states, resulting in energy shifts in the scattered light (Stokes and anti-Stokes Raman scattering)^37,63^ as seen in Fig. 1a with 1b depicting a spectra of titanium dioxide (TiO_2_) highlighting both the Stokes and anti-Stokes spectra. A vibrational mode is Raman active if it causes a change in molecular polarizability during vibration.^37,41,63^

Although inherently weak—typically only one in 10^7^ photons undergoes Raman scattering^30^—the technique benefits from minimal sample preparation, water compatibility, and high spatial resolution when coupled with microscopy.

To overcome the low signal intensity in Raman spectroscopy, enhancement strategies are often employed. Resonance Raman spectroscopy exploits electronic resonance conditions to enhance specific vibrational modes (Fig. 1) associated with electronic chromophores, often enhancing sensitivity by up to 10^6^ to 10^8^ times^28,41,42^ surface-enhanced Raman spectroscopy (SERS) achieves signal enhancement *via* localized surface plasmon resonances on metallic nanostructures, enabling detection of trace analytes down to single-molecule levels.^42,64–66^

Conventional Raman microscopes combine confocal optics with spectrometers, achieving sub-micrometer diffraction limited spatial resolution. In contrast, tip-enhanced Raman scattering (TERS) integrates the chemical specificity of Raman spectroscopy with the nanometric field confinement of scanning probe microscopy, thereby overcoming the Abbe diffraction limit.^67^ By exploiting localized surface plasmon resonances at a metallic tip apex, TERS focuses the excitation field into an ultra-small optical volume—typically below 10 nm—enabling nanoscale vibrational mapping of surfaces, interfaces, and nanostructures that remain inaccessible to conventional far-field Raman microscopy.^67,68^

However, for real-time, high-speed chemical imaging—especially in biological or functional material systems—nonlinear coherent Raman techniques such as coherent anti-Stokes Raman scattering (CARS) and stimulated Raman scattering (SRS) have become essential.^69^ CARS generates a coherent anti-Stokes signal (Fig. 1a) through the nonlinear interaction of pump and Stokes laser beams with the vibrational modes of the sample, offering improved sensitivity and directional signal emission.^70–72^ SRS, by contrast, detects the energy transfer between pump and Stokes beams as a modulation of intensity, providing quantitative information with high signal-to-noise ratios and without the non-resonant background typical of CARS.^29,70^

Coherent Raman imaging excels at rapid mapping of single specific vibrational marker bands and is especially powerful for monitoring dynamic processes, such as self-healing or phase transitions, in functional materials.^73^ These techniques are capable of visualizing molecular events with sub-second temporal resolution and micrometer spatial precision.

When deciding between IR and Raman approaches, it is important to consider their complementary nature: Raman is more effective for nonpolar and symmetric vibrations, while IR is superior for polar functional groups.^41^ This follows from the selection rules where IR bands gain intensity when a vibration changes the molecular dipole moment, whereas Raman bands gain intensity when a vibration changes the molecule polarizability. In molecules that possess a center of inversion (centrosymmetric), the mutual exclusion rule applies. That is, modes that are IR-active are Raman inactive and *vice versa*. Consequently, IR spectra are recorded as absorption transition between vibrational levels in the electronic ground state, whereas Raman signals arise from inelastic (Stokes/anti-Stokes) scattering when UV, visible or near-IR light interact with vibrations that modulate the molecule's polarizability. Practical interference factors also matter as Raman can be overwhelmed by fluorescence chromophores or trace impurities and drown out weak Raman signals under visible excitation. Remedies for this are discussed later. IR has its own hurdles as it faces strong water and CO_2_ absorption, pathlength saturation in transmission, and scattering/dispersion in rough or filled samples. In short, beyond the selection rules, the matrix, substrate, thickness, and excitation conditions often determine which method to use.

Beyond contrast mechanisms, spatial resolution regimes also differ between IR and Raman approaches. As mentioned before in the far field, mid-IR is diffraction limited to a few micrometers, whereas visible/near-IR Raman can reach about 0.3–1 µm with high-Numerical Aperture (NA) objectives. Nanoscale/near-field variants extend these limits with s-SNOM/nano-FTIR of about 10–20 nm and AFM-IR of about 10–50 nm while TERS for Raman is down to the sub-nanometer regime under optimized conditions.^50,62,74^ Collectively, nano-IR and TERS bridge the gap between far-field and nanoscale vibrational imaging, providing chemically specific maps with nanometer- to sub-nanometer-scale precision that complement conventional diffraction-limited IR and Raman microscopy.

Sampling modes likewise diverge as IR commonly uses transmission, attenuated total reflection (ATR), or Diffuse Reflectance Infrared Fourier Transform (DRIFT) geometries, while Raman typically is acquired in backscattering/confocal microscopes with point, line or wide-field mapping. From a practical standpoint, FT-IR benefits from multiplex/throughput advantages for rapid, high signal to noise ratio (SNR) spectra and imaging, whereas Raman mapping generally offers finer spatial resolution at the cost of longer acquisition times which are mitigated by fast detectors and hyperspectral/lines-scan strategies.

Therefore, a combined IR and Raman strategy often yields the most complete vibrational picture of a material. The key similarities and differences between Raman and IR spectroscopy are summarized in SI Table S1.

**Fig. 2:**
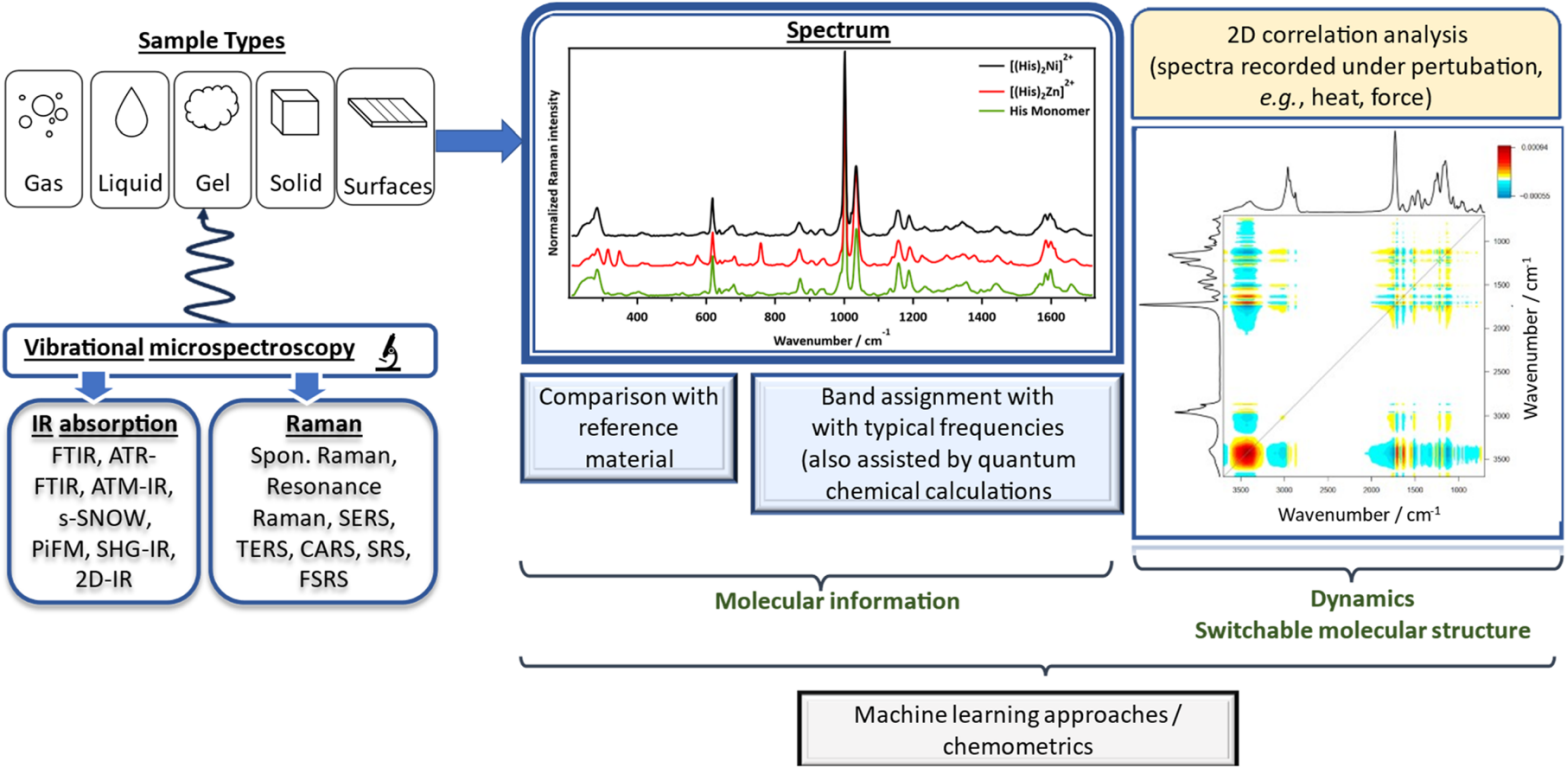
Illustration of the powerful and versatile application of vibrational spectroscopy in both research and industry, where it can be applied across all sample types and is often combined with advanced analytical and interpretative techniques—such as 2D correlation analysis and density functional theory (DFT)—to gain deeper molecular insights.

Finally, to interpret complex vibrational spectra—especially in multicomponent or dynamic functional materials—advanced computational and statistical tools are indispensable. A few illustrative examples of such tools are illustrated in Fig. 2. Density Functional Theory (DFT) calculations, which is rooted in quantum mechanics, provide a direct molecular structure-to-spectrum by computing the Hessian to obtain normal modes, and from these, evaluate IR intensities (dipole-moment derivatives) and Raman intensities (polarizability derivatives) to help assign vibrational bands.^75–77^

Similarly, 2D correlation spectroscopy (2DCOS)^78–80^ and multivariate analysis^81^ enable enhanced resolution and extraction of subtle spectral changes under perturbations such as temperature or mechanical stress. By projecting each band's perturbation driven response into a second dimension, 2DCOS separates overlapping peaks and provides insights into which signals are correlated, and which one changes first. This results in two maps: the synchronous map (in phase, real component, *Φ*(*v*_1_,*v*_2_)) provides information as to whether two bands vary together (by positive cross-peaks) or in opposite directions (negative cross-peaks), and the asynchronous maps (out of phase, imaginary components, *Ψ*(*v*_1_,*v*_2_)) gives information about the order of events.^78,80^ Following Noda's generalized rules,^78,80^ when the synchronous and asynchronous cross peaks have the same sign (both positive or both negative), it implies that *v*_1_ changes before *v*_2_. When their signs are opposite, *v*_2_ changes before *v*_1_.

Raw vibrational spectra often carry non-chemical artefacts such as baseline curvature from scattering or fluorescence, high frequency noise, cosmic ray spikes, and instruments or batch induced drifts that can mask subtle chemical changes.^82^ To interpretate signals reliably, a processing workflow of baseline correction, denoising, despiking, wavenumber alignment, and intensity normalized prior to analysis is normally done. Because these steps are time-consuming and human dependent, this is where artificial intelligence (AI) and machine learning (ML) models are implemented to help in automating and standardizing the process into a reproducible pipeline that scales to large datasets helping to redefine the entire research cycle, from the analysis of complex characterization data to the inverse design of novel functional materials with targeted properties.^83,84^ The choice of ML model is often dictated by the task and dataset size. Classical methods such as Principal Component Analysis (PCA), Support Vector Machines (SVM), and Random Forests (RF) remain robust for smaller datasets, excelling at tasks like polymer classification and identification, even for environmentally aged microplastics.^85,86^ For more complex feature extraction and regression, deep learning models are increasingly dominant. One-dimensional Convolutional Neural Networks (1D-CNNs) learn spectral patterns directly, reducing the need for manual preprocessing,^84^ while autoencoders and nonlinear manifold learning techniques (*e.g.*, diffusion maps, conformal autoencoders) create low-dimensional embeddings that have proven superior for regressing physical properties like particle size directly from spectral features.^87,88^ This progression continues with task-specific architectures like RamanNet, which are explicitly designed for spectral invariances and demonstrate state-of-the-art performance on benchmark datasets.^89^

Overall, vibrational spectroscopy, with its suite of linear and nonlinear techniques, provides a robust analytical foundation for elucidating the composition, structure, and properties of functional materials. Its integration with imaging and computational analysis continues to expand its capabilities, making it a cornerstone of modern materials characterization. For more on the theory of vibrational spectroscopy, see the Handbook of Biophotonics.^69^

### Advanced characterization through vibrational spectroscopy

**Fig. 3:**
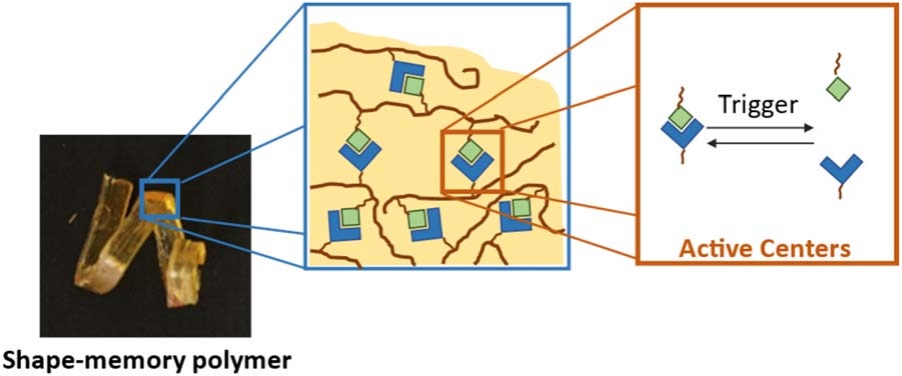
Schematic representation of a shape-memory polymer based on reversible bonds. Blue and green motifs represent the reversible bond, which can be cleaved using the corresponding trigger.

Smart materials are a subgroup of functional materials that respond to external stimuli with changes in their physical or chemical nature. Such stimuli can be temperature, pH or even light can trigger an adaptive response often yielding a reversible mechanism in the material structure or behavior. A common design principle for smart functionalized polymeric materials is the embedding of reversible molecular moieties as illustrated in Fig. 3. The exact understanding of such systems is a highly complex task, as it requires analysis of the reversible moiety, the environment and most importantly the interplay or interaction between the two entities. To gain deeper insight into the function of said materials, understanding the structure and linking it to the function is essential. To achieve this, it requires the separation of information originating from a small part of the system (the active center) and information originating from other parts of the system. However, the characterization of such integrated systems using conventional analytical methods commonly used in synthetic chemistry, such as NMR spectroscopy and mass spectrometry, presents a challenge. While these methods offer high molecular sensitivity, they often suffer from overlapping bands and low signal strength in complex environments, preventing useful access to active sites in smart functionalized materials. Moreover, these techniques can often not be utilized in highly crosslinked materials. As systems become more complex, this is an increasingly difficult task. Nevertheless, the targeted development of smart functional materials requires a precise understanding of the molecular processes occurring in the materials that are responsible for the desired functionality. As a result, many complex functional systems are still poorly understood at the molecular level at present, which makes the targeted design of such systems considerably more difficult. This therefore requires non-invasive analytical methods like vibrational spectroscopy in combination with complex evaluation algorithms.

Building on this need, and following the theoretical considerations outlined above, which highlighted the principles, capabilities, and spatial–temporal limits of modern vibrational spectroscopic techniques, we now turn to their application in the study of smart and functional materials. Modern vibrational spectroscopic methods, in combination with computer-aided spectral analysis approaches, offer a particularly powerful framework for probing the structure, composition, and dynamics of such responsive systems. In the following, we illustrate the versatility and analytical power of vibrational spectroscopy through three selected examples, before summarizing recent work on its application to the characterization of the aforementioned classes of functional materials. For clarity, we note that this review does not address surface-enhanced Raman spectroscopy (SERS), as numerous comprehensive reviews on this topic already exist, nor does it cover nanoparticle-based systems that are closely associated with SERS and plasmon-enhanced signal generation, as these have likewise been extensively discussed elsewhere.^90–94^

**Fig. 4:**
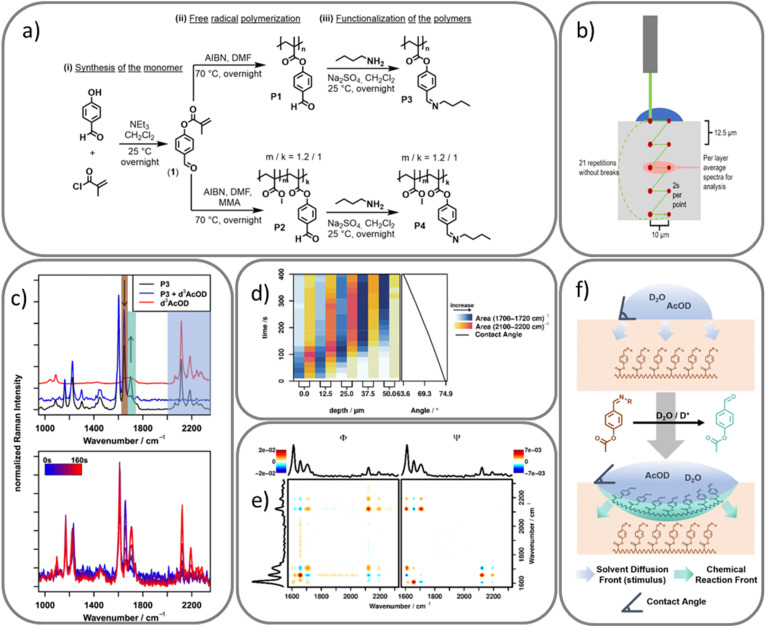
(a) Synthesis pathway of monomer 1 (4-formyl phenyl methacrylate), its polymers (P1, P2), and imine-functionalized derivatives (P3, P4). (b) Schematic depiction of the confocal Raman microscopy measurement scheme. (c) FT-Raman spectra showing imine hydrolysis in P3 upon treatment with d^3^-AcOD, and time resolved Raman spectra of P4 after surface activation. (d) Time- and depth-resolved analysis of the hydrolysed polymer (C=O band) and acetic acid influx (C–D band), alongside contact angle measurements indicating increasing surface hydrophilicity. Blue-shaded region corresponds to 1700 to 1720 cm^−1^ (aldehyde C=O) and 2100 to 2200 cm^−1^ corresponds to C–D stretching vibration. (e) 2D correlation spectra ((left) synchronous and (right) asynchronous) at 25 mm depth confirming the sequence of hydrolysis followed by acid diffusion. (f) Schematic illustration of the surface-active polymer design principle. Upon exposure to the external stimulus (D_2_O/AcOD, shown in blue), the imine side groups of the polymer (P3/P4, dark orange) undergo hydrolysis, forming aldehyde groups (mint green). Following this bond cleavage, the activating solvent continues to diffuse deeper into the polymer matrix. This results in a temporal and spatial delay between the chemical reaction front, which initiates at the interface, and the advancing solvent diffusion front. All figures are reproduced with permission.

As mentioned earlier, smart materials are engineered to respond predictably to external stimuli such as pH, temperature, or light. In a recent study, a smart polymer functionalized with imine groups was designed to undergo a switch from hydrophobic behavior to hydrophilic behavior upon exposure to acid (see Fig. 4a). This switch response raises several mechanistic questions: How can this transformation be effectively monitored? Does the acid first diffuse into the polymer before cleaving the imine bond to form aldehyde groups or does bond cleavage occur initially at the surface and subsequently facilitate further diffusion? What is the correlation between the chemical event at the molecular level and the observed macroscopic changes? And finally, how can the diffusion and switching be quantified?

To address these questions, Hniopek *et al.*^95^ employed spatially resolved Raman micro-spectroscopy as a non-destructive, *in situ* tool capable of simultaneously tracking both the diffusion of the stimulus and the chemical transformation occurring within the polymer matrix.

To do this, a mixture of heavy water and deuterated acetic acid (D_2_O/d^3^-AcOD) in the ration of 9 : 1 volume ratio was used as the pH trigger, as this will separate the vibrational bands arising from the activating mixture and those from the polymer into the so-called “silent region” of the Raman spectrum, thus simplifying analysis.

A reference Raman measurement was done using Fourier transform (FT) Raman spectroscopy by introducing a drop of the mixture onto the polymer surface initiating a clear stimulus response through hydrolyzing of the imine bonds, leading to the formation of aldehydes groups. This process was clearly reflected in the Raman spectra (Fig. 4c), which revealed a decrease in the imine C=N stretching vibration intensity at 1656 cm^−1^, accompanied by a corresponding increase in the aldehyde C=O stretching vibration at 1702 cm^−1^. Additionally, the appearance of bands at about 2100 to 2300 cm^−1^ region indicated the influx of AcOD. The presence of these bands confirms the hydrolysis process has occurred. *In situ* Raman microscopy was used then to investigate the progression of the stimulus and the response of the polymer order of diffusion by monitoring these bands under. To do this, the sample was hot pressed, embedded in an epoxy matrix, placed under the Raman microscope and a scan of the surface from 0 to 50 µm depth in 12.5 µm increment were performed and at each depth, two lateral points spaced 10 µm apart were recorded with two seconds integration time (see Fig. 4b). This procedure was repeated when the mixture was dropped on the polymer at the focus point. Consistent spectral features were observed as in the reference measurement.

Detailed analysis revealed that the spectral bands corresponding to the hydrolyzed polymer (C=O at 1702 cm^−1^) appeared first before any signal of the acetic band at about 2100 to 2200 cm^−1^ is seen at about 60 s into the measurements suggesting that bond cleavage of the imine bond to aldehyde precedes acid influx into the polymer. This finding implies that the free aldehyde groups cause the hydrophilicity of the polymer thereby facilitating subsequent penetration of the aqueous mixture.

To quantify the molecular changes, the intensities of the aldehyde C=O band and the C–D vibration at 2100 cm^−1^ were integrated and plotted (Fig. 4d). The resulting time resolved data confirmed that hydrolysis (blue-shaded region) occurred or happens earlier before the influx (yellow to red shading) across all scan depths.

Moreover, to further elucidate the sequence and correlation of these molecular events, 2DCOS was employed. As an advanced computational and statistical tools, 2DCOS enhances interpretability of complex spectral datasets, separates overlapping bands, determine the sequence of spectral events, and identifying correlation between vibrational modes by spreading spectral information over a second dimension, thereby making subtle changes more distinguishable.^78^

Following Noda's rules, 2DCOS analysis (Fig. 4e) revealed a positive correlation of the aldehyde band at 1702 cm^−1^ and the acetic band at 2100 to 2200 cm^−1^ indicating they both change (increase) in intensity together. At the same time, the hydrolyzed aldehyde band at 1702 cm^−1^ is negatively correlated to C=N unhydrolyzed polymer at 1656 cm^−1^, reaffirming that imine hydrolyzation occurs as a direct addition of the activation mixture. To determine the temporal order of events, the synchronous and asynchronous maps were compared and since the correlation signs are both consistent in each map for the pairs (1702, 2110), (1656, 2110) and (1656, 2180) cm^−1^, it conclusively confirms that the diffusion of acetic acid always happens after the polymer has been hydrolyzed. Additionally, time delays between hydrolysis and acid influx were calculated supporting this sequence of events. For numerical values, the reader is referred to the original article.^95^

Finally, one of the most valuable contributions of 2DCOS in this study was its ability to extract small and systematic changes from spectra that are otherwise difficult to detect. This was particularly useful in analyzing the very weak Raman signals of the O–D stretching bands in the 2500 to 2600 cm^−1^ region, where traditional integration methods fail due to low intensity. However, through 2DCOS, a clear meaningful correlation was revealed between the O–D and the C=O stretching bands, further confirming that bulk influx of the D_2_O also occurs after hydrolyzation of the imine.

This highlights that the combination of Raman micro-spectroscopy with advanced interpretation techniques such as 2DCOS, serves as a powerful analytical approach capable of uncovering subtle molecular dynamics as demonstrated above in smart materials.

**Fig. 5:**
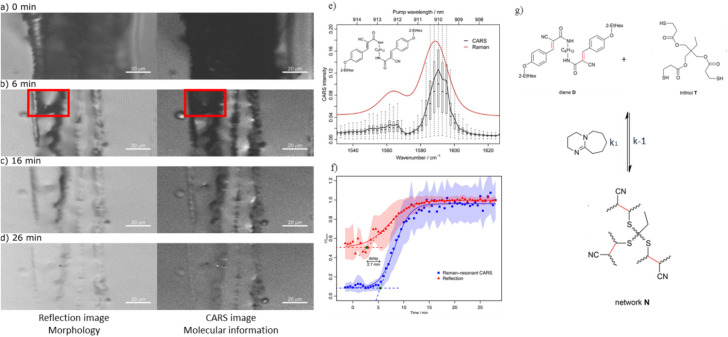
(a–d) Sequential reflection (left) and CARS (right) images of the scratch at 0, 6, 16, and 26 minutes at 60C. The red square in (b) highlights a region where healing is visibly faster in the reflection image than in the CARS image, indicating a delay between physical healing and chemical reaction. (e) The CARS and linear Raman spectra of compound D show excellent agreement, confirming that non-resonant background is negligible in the CARS imaging for this system. (f) A plot of normalized reflection and CARS intensity over time reveals a time delay (about 2.7 minutes) in the onset of healing observed by CARS compared to reflection. This suggests that molecular diffusion precedes the chemical reaction. (g) A schematic of the thiol–ene equilibrium illustrates the healing mechanism. CARS specifically tracks the alkene groups (highlighted in red), which are consumed during network formation as the material heals. All figures are reproduced with permission.

Morphology-based techniques such as scanning electron microscopy (SEM)^96^ or atomic force microscopy (AFM)^97^ have mostly been employed to study functional materials. However, these techniques alone cannot determine whether an observed transformation, such as healing, is driven by chemical reactions, diffusion processes, or even mechanical rearrangement. As such, there is a clear need for techniques that can provide real time molecular-level insights into the kinetics or these processes to capture simultaneously the structure and chemical changes as healing occurs. Conventional linear Raman spectroscopy, like the one employed by Hniopek *et al.*,^95^ lacks the temporal resolution needed to monitor rapid kinetic processes. This is where non-linear approaches such as CARS become highly advantageous. CARS offers fast imaging capabilities that enable real-time observation of molecular interactions and structural changes, particularly during dynamic processes like healing. This enables researchers to correlate macroscopic morphological recovery with molecular level parameters such as diffusion rates and reaction kinetics. This potential was demonstrated by Geitner *et al.*,^73^ who used CARS to study thermo-reversible thiol–ene bonds, which were embedded in a polymeric matrix resulting in a self-healing polymer. At temperatures above 60 °C, partial de-crosslinking of the network occurs. When the polymer is subsequently cooled, the thiol–ene reaction initiates, leading to the healing of the polymer material and restoration of its properties (Fig. 5g). The key questions arising from this study are: How was this healing process monitored? What correlation can be established between the morphological changes to the underlying molecular changes?

To effectively use CARS with high spectral clarity, a strong, distinct signal within the region of interest is needed. This minimizes interference from the non-resonant background, enabling clear spectral interpretation and chemical specificity of interest. For this reason, Geitner *et al.* compared the CARS spectrum of the –C=C– stretching region of the thiol–ene compound to its linear Raman spectrum. As shown in Fig. 5e, the two spectra closely match providing strong evidence that the observed CARS signal is dominated by the vibrational resonance and that the non-resonant background can be neglected in this study.

To monitor the self-healing process, a scratch was first inflicted into the polymer, followed by heating to 60 °C to trigger healing. CARS imaging was then performed using the –C=C– stretch-vibration region, which enabled spatial and temporal monitor of free alkene groups. Simultaneously, laser reflection images were used to monitor morphological evolution of the scratch. By comparing these two imaging modalities, they observed that the CARS image representing the chemical reaction progress filled up more slowly than the reflection image which captures the physical filling of the damaged area. This is illustrated in Fig. 5b, red box. Time resolved analysis (Fig. 5f) revealed that the self-healing process began approximately 2.7 minutes earlier in the reflection images than in the CARS image. This time delay was calculated for every pixel by comparing the normalized, time dependent profiles of the reflection and Raman resonant CARS signals. This discrepancy occurs because reflection imaging captured only the diffusion of molecules into the scratch area, while CARS specifically capture or monitors the reaction itself. As a result, the delay in the CARS image reflects the time required for the reaction to occur even though the diffusion has already started. These findings demonstrate CARS offers a more detailed understanding of the molecular-level insight into the kinetics reaction process within the scratch providing invaluable insights for fine-tuning the properties of the self-healing polymers.

While both linear and non-linear Raman spectroscopy are highly effective for monitoring vibrational modes and subtle molecular changes, they are less sensitive to highly polar functional groups like urea, amides, carboxylic acids, alcohols and nitriles. These groups contain bonds with large electronegativity differences (*e.g.*, N–H, O–H, C=O, C≡N) which produces strong dipole moments and are, thus, more IR-active than Raman-active.^98^ Raman scattering primarily depends on changes in molecular polarizability rather than dipole moment, making it less effective for tracking vibrations dominated by highly polar bonds. Therefore, integrating infrared (IR) spectroscopy offers complementary sensitivity, enabling a more comprehensive vibrational analysis of the material's chemical structure and dynamic behavior.

It is for this reason Schäfer and Kickelbick^99^ employed IR spectroscopy to investigate a novel functional poly(butyl methacrylate) (pBMA)-based hybrid materials, which was capable of self-healing. The reversible Diels–Alder (DA) of maleimide and furan was utilized for the crosslinking of the polymer networks. In addition, hydrogen bonding motifs, based on urea and amides, respectively, have been incorporated into the polymers.

**Fig. 6:**
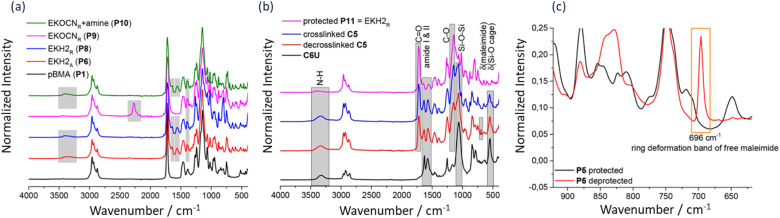
Illustration of the IR spectral analysis of functionalized pBMA-based polymers. (a) Comparison between the IR spectra of pure pBMA and functionalized one-component polymers EKH 2 (b) FTIR spectra of a novel functional poly (butyl methacrylate) (pBMA)-based hybrid intrinsic self-healing polymer systems incorporated with Diels–Alder (DA) cross-linking moieties as well as self-assembling supramolecular hydrogen bonding units. (c) Confirms successful deprotection of polymer P5 through the appearance of a characteristic maleimide deformation band. All figures are reproduced and/or adapted with permission.

This study is a collective approach that builds upon a previous finding within their group and the research group of Raquez^100^ on one-component DA self-healing films. The former observed that smaller inorganic molecular building blocks potentially led to faster healing and as such Diels–Alder (DA) systems that are spherosilicate or silsesquioxane exhibit more efficient self-healing than those using SiO_2_ nanoparticles. The latter found that adjusting the ratio of hydrogen bonds to DA moieties led to a free-standing self-healing film. IR spectroscopy was utilized in confirming the formation of the functionalized polymer and in analysis its self-healing ability and the DA bonds in the polymer, which are thermally triggered. The presence of urea groups was confirmed through characteristic IR absorption bands at 3400 cm^−1^ (N–H stretching), 1637 cm^−1^ (amide I, C=O stretching), 1558 cm^−1^ (amide II, C–N stretching), and 1392 cm^−1^ (C–N–C stretching, N–H deformation, CH_3_ deformation) (Fig. 6a). Furthermore, the presence of spherosilicates contained in the urea group was also confirmed by distinctive Si–O–Si stretching and cage deformation vibrations at 557 cm^−1^ in the FTIR spectra of Fig. 6b.

FTIR analysis also confirmed the reversibility of the polymer network. Upon thermal treatment at 120 °C for 30 min, a new ring deformation band appeared at 696 cm^−1^, indicating the regeneration of free (yet intact) maleimide groups verifying deprotection and thermal reversibility. Subsequently, this band disappeared after re-crosslinking at 70 °C for 20 hours, indicating successful crosslinking by the DA cycloaddition within the polymer (Fig. 6c). In addition, FTIR confirmed hydrogen bonding interactions within the bulk polymer matrix, with broad absorption bands at 3300 and 3336 cm^−1^ for the respective polymers (Fig. 6a and b). These were attributed to a dynamic hydrogen bond network formed between the filler and matrix, which is essential for the miscibility and homogeneity of the composite. Moreover, within the inorganic component of the material, spontaneous hydrogen bond formation occurred throughout the matrix and its surface. Owing to its glue-like effect, this bonding behavior provides immediate contact at cut interfaces, thereby enhancing the material's self-healing effectiveness.

This in-depth review of the three papers highlights how both label-free linear and non-linear vibrational spectroscopy techniques can be effectively applied to study functional materials. In the following chapter, we further explore selected examples of smart functionalized materials and discuss how vibrational spectroscopy has been employed to characterize their development, functional behavior and dynamic responses.

### Self-healing polymer

Self-healing materials are functional materials with the ability to autonomously regenerate and repair itself after sustaining damages. This concept is inspired from biological systems, such as the human skin, which exhibits remarkable healing abilities by initiating complex cellular and molecular mechanisms to rebuild damaged tissue after cuts,^101–103^ or the merging and healing of fractured bones,^104,105^ and even the repair of damaged DNA at the molecular level.^106,107^ Another fascinating source of inspiration is the axolotl (*Ambystoma mexicanum*), also known as the Mexican salamander, which is known for its extraordinary regenerative capabilities, including the ability to grow entire limbs, parts of its spinal cord, portions of its heart, and even segments of its brain.^108–110^

This biologically inspired self-healing concept, when applied to artificial materials, aims to prolong their lifespan, improve safety, and reduce maintenance costs while minimizing environmental impact. What makes these materials even more exciting is their ability to restore their overall functions such as conductivity, elasticity, and mechanical strength and other essential performance metrics.^24^ As a result, self-healing materials have found applications in diverse industries, including aerospace, electronics, protective coatings, and civil engineering (*e.g.*, self-healing concrete^111^ and asphalt pavements^112,113^).

**Fig. 7:**
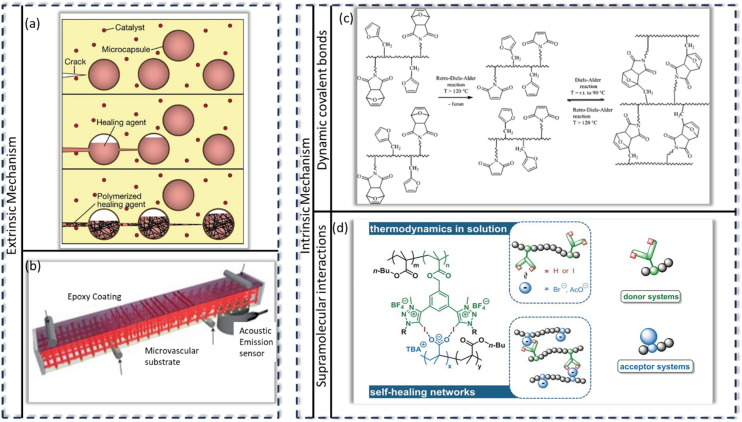
Extrinsic and intrinsic self-healing mechanism. Extrinsic approaches: (a) microcapsules containing healing agents that raptures and migrates to crack site and polymerize (often *via* an embedded catalyst); (b) *via* microvascular networks that deliver healing agents to crack site on demand. Intrinsic approaches: (c) reversible covalent chemistry such as Diels–Alder/retro-Diels–Alder reactions; (d) supramolecular interactions such as halogen bonding. All figures are reproduced with permission.

The field of self-healing polymers has come a long way since White *et al.*,^114^ in 2001, introduced the first self-healing polymeric material, which incorporated microcapsules containing a healing agent dispersed within an epoxy matrix as shown in Fig. 7a. This early model achieved self-repair by releasing the healing agent upon damage, which then polymerized to fill cracks and restore about 75% of its intrinsic toughness property. Since this pioneering work, researchers have explored and developed a wide range of self-healing polymer systems, moving from these basic microencapsulation approaches to more sophisticated intrinsic mechanisms. Self-healing polymers operate primarily through two distinct mechanisms: intrinsic and extrinsic mechanisms.^24,25,115^ Extrinsic self-healing mechanisms rely on healing agents, often encapsulated in microcapsules, which are released upon damage.^114^ The encapsulated agents then migrate to the crack site and interact with a catalyst or curing agent within the polymer matrix to repair cracks. Other extrinsic self-healing systems uses vascular networks to transport the healing agents to damaged areas which enables multiple cycles of healing.^116^

Fig. 7b depicts the microvascular network architecture. In contrast to this is the intrinsic self-healing mechanism that utilizes reversible mechanism or interactions within the polymer matrix itself. They incorporate the integration of reversible cross-linking capabilities including reversible covalent bonds, such as Diels–Alder and retro-Diels–Alder reactions^117–119^ (Fig. 7c); dynamic covalent bonds, such as disulfide exchanges^120–122^ and acylhydrazone exchanges;^123–126^ and non-covalent interactions such as hydrogen bonding,^127–129^ halogen bonding^130^ (Fig. 7d), metal–ligand interactions,^131–133^ and ionic interactions.^134–136^

To understand the microscopic and chemical mechanisms behind these self-healing processes, label-free vibrational spectroscopy techniques, particularly Raman and infrared (IR) spectroscopy, have become essential tools. As previously mentioned, label-free vibrational spectroscopy allows for a non-invasive, real-time analysis of polymer structures and provides detailed insights into the molecular and structural changes that occur during self-healing. Unlike the traditional labelling techniques, which may alter the material's inherent properties, label-free vibrational spectroscopy preserves the original chemical structure and healing properties of the polymer.

**Fig. 8:**
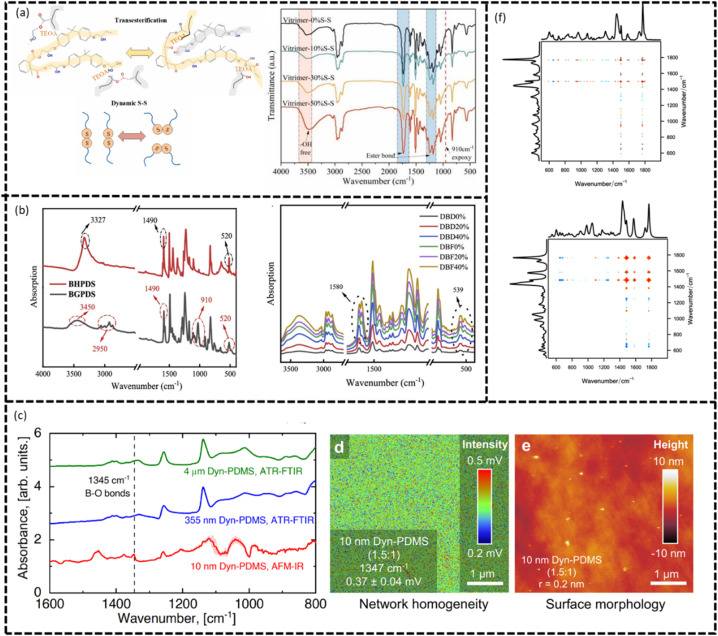
(a) Mechanisms of transesterification and dynamic disulfide bonding, along with fully cured epoxy vitrimer resins. (b) FTIR spectra of precursors (BHPDS, BGPDS) and disulfide-based self-healing epoxy resin. (c) Infrared spectra revealing characteristic B–O band at 1330–1345 cm^−1^ for dyn-PDMS (1.5 : 1) films of varying thickness. (d) For the 10 nm thin film, AFM-IR mapping confirmed the absorbance intensity distribution (0.2–0.5 mV scale), while (e) surface morphology imaging displayed height variations from −10 to +10 nm. (f) Top – Synchronous 2D Raman correlation spectrum of polymer recorded between 110–160 °C (10 °C steps) in the 500–2000 cm^−1^ region. Bottom – Corresponding synchronous 2D Raman correlation spectrum from DFT-calculated Raman data for the same temperature range. All figures are reproduced with permission.

Vibrational spectroscopy is highly sensitivity to molecular vibrations and is particularly useful for tracking bond cleavages and formation of bonds during the healing process in dynamic systems. This was demonstrated by Liu *et al.*^137^ who developed a vitrimer epoxy resin incorporating double-dynamic bonds – disulfide (S–S) bonds and esters, which can show an exchange in the presence of a catalyst – within a crosslinked network. Using IR spectroscopy, they monitored chemical bond cleavages and formations during the self-healing process by analyzing characteristic absorption peaks. The disappearance of the epoxy group peak at 910 cm^−1^ (Fig. 8a) indicated group opening and subsequent reaction with the curing agent, resulting in the emergence of strong peaks in the 1210–1163 and 1750–1735 cm^−1^ region, corresponding to C=O and C–C(=O)–O stretching vibrations, which confirms the formation of ester-based bonds and successful crosslinking. They also found that increasing the number of disulfide bonds improved the resin's dynamic properties by boosting the repair efficiency and its ability to handle extensive damage.

Similarly, Zhang *et al.*^138^ developed self-healing epoxy insulating materials for electrical equipment by integrating disulfide bonds into the resin matrix. Using Fourier Transform (FT) IR spectroscopy, they confirmed the presence and effects of disulfide bonds, identifying characteristic peaks at 539 cm^−1^ (S–S bonds), 1580 cm^−1^ (C–S stretching), and 3500–3000 cm^−1^ (hydroxyl groups). Their study showed that optimal amount of disulfide bonds enhanced mechanical properties like tensile strength and elongation, while excessive amounts reduced heat resistance, electrical characteristics, and repair efficiency due to saturation effects. The optimal resin achieved a repair efficiency of 81.23% and effectively mitigated electric-dendrite growth and damage. This is depicted in Fig. 8b.

Advanced systems, such as dual-network and supramolecular polymers, further highlight the versatility of vibrational spectroscopy as a powerful tool for capturing the simultaneous occurrence of multiple bonding interactions, including metal–ligand coordination or interactions. This capability was been demonstrated by Baetz *et al.*^139^ and Meurer *et al.*,^140^ who developed interpenetrating metallopolymers networks (IPNs) with varying crosslinking densities and different polymers backbones. To validate the formation of the desired complexes, Fourier Transform Raman (FT-Raman) spectroscopy was employed as a diagnostic tool to identify markers specific to their metal–ligand complex. Subsequently, the presence of these marker bands in the synthesized IPNs confirmed the successful formation of the targeted complexes, which demonstrates the critical role of vibrational spectroscopy in validating complex molecular designs.

Temperature-dependent vibrational spectroscopy coupled with advanced analytical techniques have significantly advanced the understanding of self-healing materials by elucidating the thermal stability and kinetics of thermally activated healing mechanisms. In particular, temperature-controlled Raman studies have been instrumental in this progress.

Another example from Geitner *et al.*^141^ utilized two-dimensional Raman correlation spectroscopy (Fig. 8f) to monitor molecular structural changes during temperature-induced self-healing in polymers based on the Diels–Alder reaction. Their study focused on the temperature range from 110 °C to 160 °C, identifying specific C=C stretching vibrations at 1501, 1575, 1585, and 1600 cm^−1^ as key marker bands for the healing process. These vibrations are directly associated with the reversible (retro-Diels–Alder) reaction between furan and maleimide, which serves as the underlying mechanism for self-healing. At elevated temperature, the six membered ring structure in P(DAP) undergoes cleavage, leading to increased mobility of the macromolecules enabling the polymer chains to migrate to damage sites, effectively filling the gaps. Subsequent, upon cooling, the Diels–alder reaction reactivated, resulting in the formation of the molecular linkages allowing the polymer to repair itself and restoring its structural integrity. The presence or absence of the aforementioned marker bands at varying temperatures provided valuable insights into the dynamics of bond formation and dissociation during the healing cycle. However, because these changes were subtle, the use of two-dimensional Raman correlation spectroscopy was crucial. This advanced technique enabled the simultaneous analysis of multiple molecular vibrations, offering a comprehensive view of the structural transformations occurring during the heating and cooling processes. Using these analysis techniques, they were able to differentiate that the bands at 1600 and 1585 cm^−1^ are increasing while the bands at 1575 cm^−1^ are decreasing in intensity during the temperature increase.

Beyond bulk spectral analysis, AFM-IR offers unique advantage of chemical mapping at the nanoscale. By coupling the spatial resolution of AFM with the molecular sensitivity of IR absorption, this technique enables direct visualization of how functional groups, particularly polar moieties, are distributed within the microdomains of self-healing polymers. Building on this capability, Jingcheng *et al.*^142^ employed AFM-IR to investigate ultrathin vitrimer films coatings, where conventional ATR-FTIR lacked sufficient sensitivity due to its limited film thickness. These nanoscale PDMS vitrimers, free of perfluorinated compounds, demonstrated durable hydrophobicity and even superhydrophobicity, with self-healing behavior arising from dynamic strand exchange. This dynamic behavior is a crucial feature since surface defects can significantly reduce coating lifetimes. Their study demonstrated that even in 5 nm dyn-PDMS layers, AFM-IR could reliably detect characteristic B–O vibrational modes, providing nanoscale evidence that the dynamic boric acid-PDMS networks remain intact after thermal annealing (Fig. 8c–e). This highlights the crucial role of AFM-IR in probing the chemical integrity of self-healing polymers systems at thickness far below the reach of traditional spectroscopic methods.

### Shape memory polymers

**Fig. 9:**
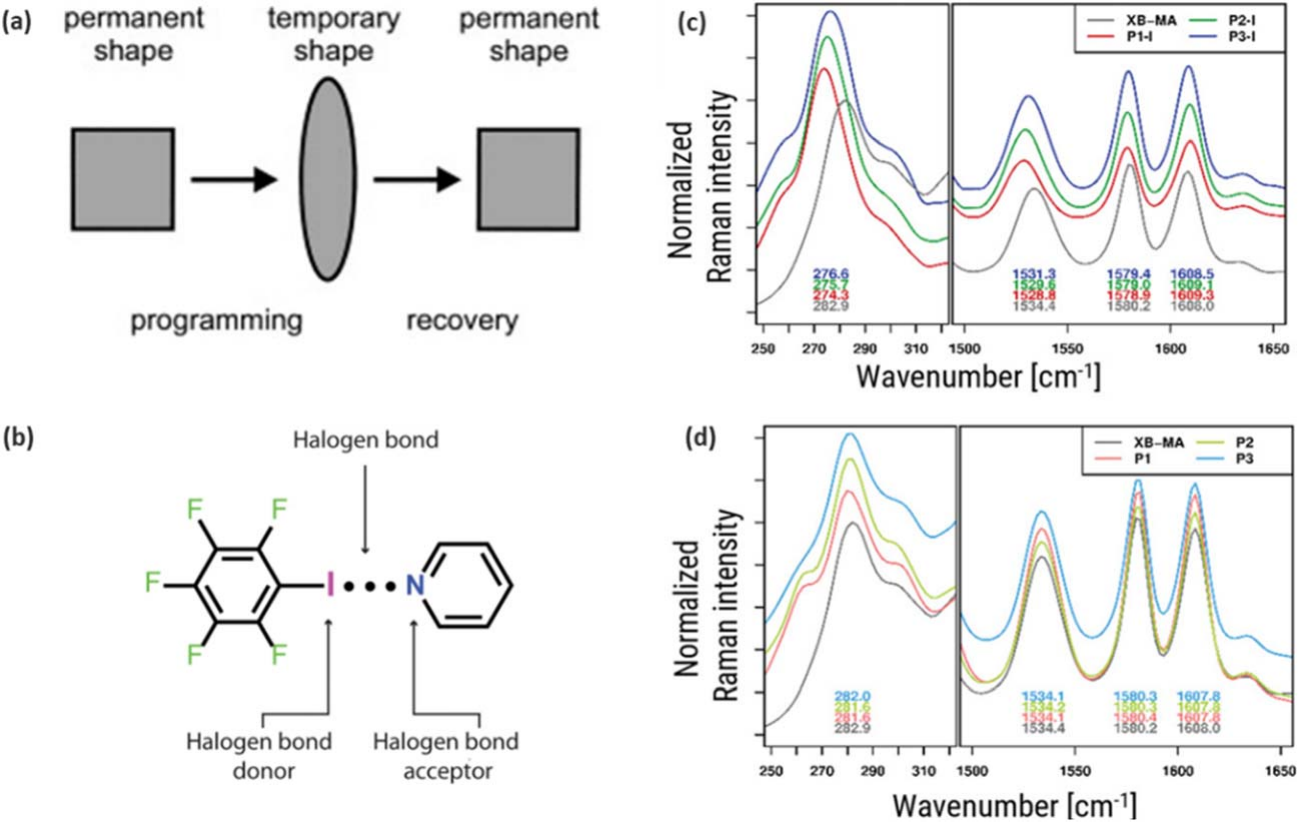
(a) Operation steps of the shape memory polymers (b) vibrational modes of C–X bonds in halogen bonded polymer with focus on the I⋯N interactions. (c) and (d) Raman spectrum of 3D-printable shape memory polymers based on halogen bonding to confirm molecular structure of the polymer. All figures are reproduced with permission.

While self-healing materials, as discussed above, primarily focuses on repairing damage by restoring molecular integrity and mechanical properties, shape memory polymers, SMPs, offer a unique ability: the ability to recover their predefined original shape when activated by specific stimuli, such as light, heat, or even an electric field.^143–145^ SMPs operates through two steps – programming and recovery – which is depicted in Fig. 9a.^146^ This remarkable property stems from their unique molecular architecture, which typically consists of two distinct phases: the hard and soft segment. The hard segment is responsible for maintaining the permanent shape, while the soft (reversible molecular switches) segment is responsible for its temporary shape.^144,146^ SMPs are categorized based on the type of stimulus required to trigger their shape recovery. Examples include thermoresponsive SMPs,^147,148^ pH-responsive SMPs,^148^ light-responsive SMPs,^144,148^ electrical responsive SMP,^148^ and magnetic responsive SMPs.^148,149^ Due to their versatility, SMPs have found wide applications across various industries. In the biomedical field, they are used in areas such as minimally invasive surgery and implantable devices. Similarly, in aerospace and automotive industry, sectors, SMPs contribute to innovations like self-deploying structures and adaptive components. Moreover, they play a critical role in sensors, actuators and the robotics industry.^150^

To further advance research into SMPs, detailed insights into the understanding of their functional, molecular, and structural mechanisms are essential. This is also where label free vibrational spectroscopy can provide valuable insights. Indeed, several studies and publications have explored this promising avenue, offering new perspectives on the development and optimization of SMPs. Here, we discuss a few of the publications where label free vibrational spectroscopy has been applied to study SMPs.

In the development of novel functionalized polymers, it is crucial to be able to verify whether the desired chemical reactions have occurred or if specific molecular structures have been successfully formed. One approach for this is Raman spectroscopy. For instance, Meurer *et al.*^151^ employed Raman spectroscopy to investigate and confirm the molecular structure of 3D-printable shape memory polymers that are based on halogen bonding. It is well established that halogen bonds form due to the charge-transfer effect, leading to partial population of the σ*(C–X) orbitals and a consequent weakening and lengthening of the C–X bond.^152^ Using the characteristic marker band of the C–I stretching vibration at 283 cm^−1^, they identified redshifts of about 6 to 8 cm^−1^ in ionic polymers, proving clear evidence of halogen bond formation (Fig. 9c). By contrast, non-ionic polymers exhibited shifts of only about 1 cm^−1^. This suggests that the magnitude of these shifts is directly correlated with the strength of the halogen bonding.

Similarly, Guo *et al.*^153^ utilized Raman spectroscopy to explore the vibrational modes of C–X bonds in halogen-bonded polymer complexes, with specific focus on I⋯N interactions (depicted in Fig. 9b). This study underscores the ability of Raman spectroscopy to detect redshifts in C–X vibration within the 100–400 cm^−1^ range upon halogen bond formation, reflecting weaker bond force constants and the n → σ* nature of I⋯N interactions. Moreover, Raman spectra distinguished XB from non-XB interactions, such as F⋯F, C–H⋯π, π–π_F_ and π_F_–π_F_, while revealing unique spectral features like doublets in asymmetric C–I bonds or singlets in type II XB systems. This capacity to unravel molecular interactions and structural changes demonstrates the indispensable role of Raman spectroscopy in designing advanced SMPs and in correlating halogen bonding with critical properties like mechanical performance.

Raman spectroscopy has also been employed to validate the formation of metal complexes by identifying characteristic marker bands unique to specific complexation processes and further confirming their presence within SMPs incorporating these polymers.^23,139,154^

Building upon these advances, Defize *et al.*^155^ introduced an innovative approach to developing functionalized light-induced SMP. By incorporating coumarin-based photo-cross-linkable units into poly(ε-caprolactone) (PCL) networks, they enabled precise, light-induced control over the polymer's properties. Raman spectroscopy was employed to investigate the relationship of the shape memory properties to the degree of coumarin dimerization under light irradiation. The –C=C– is highly Raman-active due to its strong polarizability changes during vibration.^98^ Specifically, the characteristic Raman band for the –C=C– bond in coumarin appears at 1562 and 1617 cm^−1^. Under irradiation, these bands showed a progressive decrease in intensity, providing clear evidence of the dimerization process. Furthermore, the spectra were analyzed quantitatively to determine the extent of coumarin conversion, establishing a direct link between the photochemical reaction and the polymer's shape memory properties. of the coumarin band intensity of these characteristic bands. This detailed spectroscopic analysis highlights the critical role of vibrational spectroscopy in optimizing light-responsive SMPs.

Due to the polar nature of functional groups such as imide C=O, C–N, and aromatic ring vibrations, IR spectroscopy is often preferred because it is highly sensitive to polar bonds and detects strong dipole moment changes during vibrations.^98^ In this context, Qiu *et al.*^156^ used infrared (IR) spectroscopy to confirm a facile way for synthesizing high-temperature shape memory copolyimides with controllable glass transition temperatures (*T*_g_) ranging from 183 to 230 °C. These copolyimides derive from the copolymerization of 4,4′(4,4′-isopropylidenediphenoxy)bis(phthalic anhydride) (BPADA) with diamine combinations of 1,3-bis(3-aminophenoxy)benzene (BAB) and 4,4′-bis(4-aminophenoxy)biphenyl (BAPB). Their study aimed to understand how the diamine components influence the *T*_g_ of these SMPs. Characteristic absorption bands in the IR spectra revealed the presence of imide functional groups, which are essential for the copolyimides' shape memory properties. Specifically, the asymmetric stretching vibration of the carbonyl (C=O) group appears around 1775 cm^−1^, while the symmetric stretching vibration was observed near 1713 cm^−1^. Additional bands around 1375 cm^−1^ correspond to the C–N–C bond, and those between 720 and 740 cm^−1^ were attributed to the bending vibration of C=O. The study found that these thermally stable copolyimides demonstrated strong shape memory performance, primarily due to the large difference in storage modulus between their glass and rubbery states, which contributed to their high shape fixity. Additionally, strong π–π interactions and substantial chain entanglements contributed to their high shape recovery. The relationship between *T*_g_ and diamine components conformed with the Fox equation, providing a reliable method for designing high-performance SMPs with tailored properties.

**Fig. 10:**
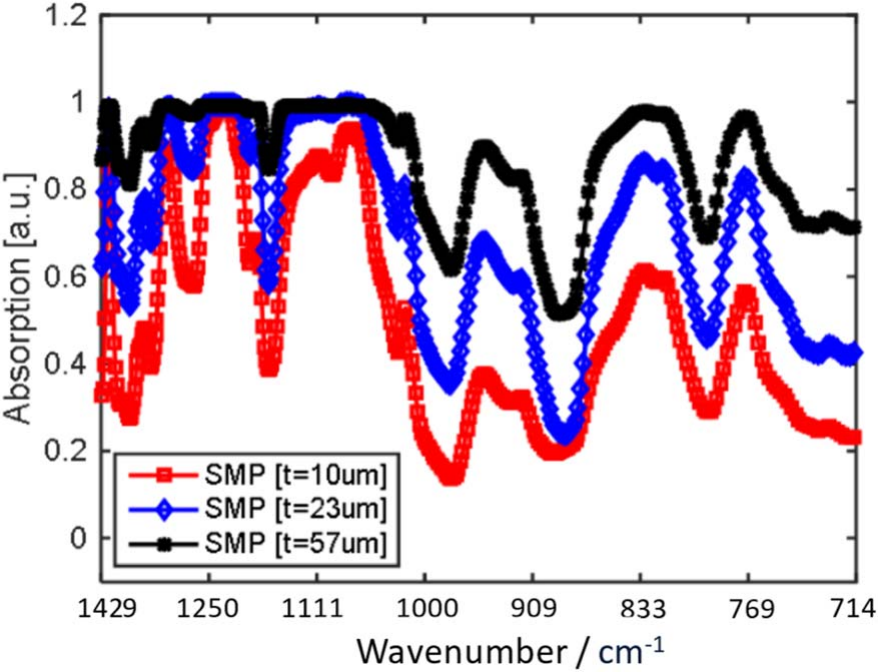
Absorption spectra of the shape memory polymer (SMP) membranes were analyzed using SMP sheets with thicknesses of 10 µm, 23 µm, and 57 µm across the wavelength range of 7 µm to 14 µm. Figure reproduced and/or adapted with permission.

Another fascinating study by Adiyan *et al.*^157^ utilized highly sensitive uncooled resonant infrared sensors that are based on thermo-responsive shape memory polymers. These thermal detectors transduce IR radiation by measuring temperature-dependent physical property and in this case, heat. Integration of the SMP in the sensor increases the temperature coefficient of the resonant frequency (TCF) making it sensitive to changing its mechanical property. TCF plays a crucial role in enhancing the Noise Equivalent Temperature Difference (NETD), which is a significant metric for determining the sensitivity of IR sensors. The sensor primarily operates by inducing a temperature change on the SMP resonator from the incident IR radiation of the target leading to a change in the Young's modulus of the SMP material which then shifts the mechanical resonance frequency of the resonator that can be detected using a frequency readout scheme and then can be converted back to a temperature change. The SMP of the sensor must effectively absorb IR radiation to function optimally. To evaluate this, an FTIR spectrometer was used to characterize the absorbance of the SMP sample. The SMP with thickness of 10 µm, 23 µm, and 57 µm were found to absorb about 48%, 69% and 84% of the IR radiation, respectively, within the 7–14 µm spectral range as illustrated in Fig. 10. At the 10 µm thickness, the SMP is already a good absorber in thus was used for the completion of the study. Furthermore, their study also revealed that SMP sensors maintain a very high performance even under atmospheric pressure, which exhibits minimal degradation compared to vacuum conditions. This is attributed to the sensor's high sensitivity and inherent thermal isolation properties.

### Photocatalytic functional materials

While photochemistry and spectroscopy date further back, the intentional study of light–matter interactions began around the late 18^th^ to early 19^th^ century. Its upward journey continues today and while in some areas the research is adept as with every area there is still space for growth. One of the earliest works of water splitting by Fujishima and Honda in 1972,^158^ aided in laying a foundation for photocatalysis. While one often thinks of water splitting when it comes to photocatalytic reactions, the field extends far beyond that. It spans across many areas such as food and safety, healthcare, automobile and transportation to name a few.^159,160^ Here, we delve into examples where spectroscopic methods were utilized to investigate and characterize materials and compounds developed to improve everyday life. As mentioned earlier, spectroscopic studies provide an essential foundation for understanding molecular interactions of the components of a system. Techniques such as linear and non-linear Raman spectroscopy, UV-Vis, IR and XPS have played a pivotal role in understanding these mechanisms in bulk, surfaces and interfaces.^161,162^

For instance, in addition to finding CO which is a dominant product of an electrochemical reaction of CO_2_ reduction reaction (CO_2_RR) on Ag in the 1800–2200 cm^−1^ region in an *in situ* SERS study, C3 products (propane, acetone) and C4 (butanol) were also discovered while conducting Ag-catalyzed CO_2_RR. Two notable changes employed in this work were the omission of an applied potential at the surface of Ag NP in visible light, which was achieved through higher sensitivity with 200 ms temporal resolution and nanoscale volume size, and secondly, under the focused plasmonic excitation with an intensity of ∼108 W m^−2^, thereby achieving an average time between absorbed photons of ∼700 fs which is shorter than electron–hole recombination (1 ps). Due to CO_2_RR requiring a counter oxidation reaction, they were able to confirm this by examining the SERS spectra and finding oxygen and hydrogen peroxide both which results from water oxidation from data collected both experimentally and calculated (DFT calculations of normal (^12^C) and heavy (^13^C) isotopes in the gas phase at 298.150 K and 1 atm) based upon isotope labelling when applied to nanoscale probing, supported the verification that the species detected are the intermediates and products of the catalytic reaction, rather than unrelated contaminants. This led to the inference that C–C coupling on plasmon-excited NP surfaces is a favored outcome.^161,163^

In another example, graphene's characteristic G and 2D bands were studied using Raman spectroscopy in both single sheets and bulk forms of graphene. Raman spectra revealed that while graphene signals remained unaffected by different substrates, there were, however, process-induced defects and stress in graphene by depositing a gate insulator oxide. While the aim was not to damage the graphene layer nor change its electrical properties, this was done and observed using Raman spectroscopy and imaging. The changes that occurred from the oxides are those *via* doping, defects, and various mechanical deformations. Upon adding 5 nm SiO_2_ lead to the immediate appearance of a D′ peak at 1620 cm^−1^ appears which can be a result of deposition or SiO_2_ and graphene interaction producing vacancies, dislocation and/or dangling bonds. When in bulk these peak decreases and a similar pattern is seen when annealing which was then done to repair the damage, while the defects are not seen in bulk it is important to note here that annealing does indeed repair the damage from deposition. A shift to higher frequencies of all Raman bands is also seen with the temperature increase and was attributed to compressive stress exerted on graphene from SiO_2_ as it becomes denser on annealing. This was the first study of its kind in this regard. The Raman images also show a change in intensity upon deposition of SiO_2_ of the D band indicating that the damage is seen only at thin layers of graphene deposition, and contrary to this the G band showed no changes.^162^

Another notable investigation utilized X-ray photoelectron spectroscopy (XPS) studies, FTIR, Raman spectroscopy and UV-Vis to confirm the formation graphene under UV radiation. This proved to not only be a green chemistry method in producing reduced graphene oxide dispersions from graphene oxide nanosheet *via* UV-irradiation, but also using polyvinyl pyrrolidone (PVP) functionalization produced a stable colloidal suspension of reduced graphene oxide that is homogeneous. These were confirmed by the UV-Vis redshift of the K-band from 230 nm to 260 nm and disappearance of the B-band at 300 nm after 48 h indicating highly conjugated structures of graphite had been formed, as well as a similar redshift seen at the K-band and decreased B-band intensity over time upon PVP-functionalization. Likewise, the FTIR spectra before and after UV reduction, the strongest vibrational absorption bands of C–OH around 3430 cm^−1^ (O–H stretching vibrations) and COOH became weak, and the vibration band of epoxy groups disappeared. While the Raman spectra of carbon materials have a D (sp^2^ C-str.) and G (sp^3^ C-str.) band whose intensity ratios are an indicator for disorder, in graphene the G mode at 1350 cm^−1^ is the first order scattering of the E_2g_ phonon of sp^2^ C atoms at 1575 cm^−1^ and the D mode arises from a breathing mode of *k*-point photons of A_1g_ symmetry at 1350 cm^−1^ showed an increased D/G ratio indicating defects or edge areas. The Raman spectra also showed the 2D band in the graphene oxide and UV-reduced samples indicating single and multilayer nanosheets were present. Lastly, the XPS data shew a sharp decrease in oxygen's absorbance peaks after UV reduction indicative that epoxide and hydroxyl groups were removed. In all graphene was formed using UV irradiation without a reducing agent or photocatalyst.^164^

Congo red (CR) a known carcinogenic substance was used as a contaminant to investigate wastewater management through the potential of reduced graphene oxide (rGO) doped with synthesized porphyrin (5,15-bisdodecyl porphyrin, C12P) to produce a reduced graphene oxide (rGO–P nanocomposite). This nanocomposite's ability to degrade CR *via* photocatalysis and antimicrobial properties was investigated having Raman spectroscopy and UV-Vis amongst the methods employed in proving graphene's effectiveness in wastewater management. The UV-Vis spectra showed a π → π* transition of C–C bonds at 284 nm indicating a highly conjugated structure was made, and a weakly detected n→π* transition at 224 nm from functional groups with oxygen, furthermore upon functionalization two blue shifts, one at 402 → 347 nm and the other at 502 → 477 nm. This shift was attributed to the interaction between C12P and rGO due to covalent interaction or π–π stacking.^165,166^

Real life applications of the above are the use of bulk graphene as gates in p–n junctions and the results of CO_2_RR can be used in further development of CO_2_ capturing of greenhouse gases. Graphene has the potential to be applied to numerous areas from cosmetic materials and water treatment to photocatalysis systems due to its ability to reduce electron–hole recombination, furthermore since graphite is quite abundant therefore it is environmentally friendly and cost effective.

### Stimuli responsive hydrogels

Hydrogels are crosslinked polymers that have remarkable capacity to retain water. Superabsorbent hydrogels, in particular, can retain water hundreds of times their weight, which is highly dependent on the availability of free hydrophilic groups and the degree of crosslinking during synthesis.^167^ Crosslinking can occur *via* two mechanisms: physical crosslinking which involves reversible interactions such as van der Waals forces and hydrogen bonds or chemical crosslinking, in which irreversible covalent bonds are formed. Due to hydrogels dual origin, naturally and synthetically, this enables hydrogels to be used across a vast spectrum of applications.^168^

Raman spectroscopy was utilized to better understand the degree or effect crosslinking. For example, poly(vinyl methyl ether) (PVME) was studied in this context. This polymer features an interesting property, since it has a lower critical solution temperature (LCST) at 37 °C. At this temperature, the water soluble polymer becomes insoluble and precipitates. This temperature enables the hydrogel to be a good candidate for medical applications. In a volume phase transition (VPT), water is rapidly expelled pulled out from collapsing polymer network. However, previous studies have identified that there is an overlap of spectra when measuring IR, which led to difficulty in the proper assignment of CH bands between 2800–3000 cm^−1^. To address this, polarized Raman spectroscopy was employed to deconvolute the bands. Parallel and perpendicular polarization were done and resulted in the following observations: at 2893 and 3000 cm^−1^ had a low intensity for perpendicular polarization, the symmetric CH bands at 2842 and 2922 cm^−1^ showed a significant decreased as well. A decrease in relative intensity of the band at *ca.* 2951 cm^−1^ and 2998 cm^−1^ due to antisymmetric stretching was also observed. Overall, a decrease in Raman intensities was observed when comparing dry hydrogels to their swollen counterparts. Further studies into the effect of crosslinking concluded that high crosslinking degree leads to coil-to-globule transition of PVME chain, resulting in a stiffer network with slower reactions to temperature changes. Moreover, the crosslinking process may reduce the number of hydrophilic methoxy groups, rendering the gel more hydrophobic. This behavior was confirmed by observing the kinetics based upon a shift in the *ν*_s_(CH_3_) band at 2842 cm^−1^ due to symmetric stretching with an increase in crosslinking. Difference spectra of Raman measurements repeated at varying temperatures between 39–50 °C also revealed that this hydrophobic behavior is also seen once above VPT, the gel is above the LCST and the structure of water present in the gel is less distressed.^168–170^

In another study, vibrational spectroscopic techniques were applied to analyze a biodegradable hydrogel derived from cellulose extracted from rice hull. The hydrogel was synthesized from carboxylate cellulose with trichloroacetic acid grafted with acrylic acid and cross-linked with four cross-linkers through the esterification reaction. The objective was to evaluate its water absorption capacity and cytotoxicity on human embryonic kidney cells. FTIR spectroscopy highlighted differences between the rice hull and cellulose with bands at 1725 cm^−1^ (*ν*(C=O) of aliphatic ketone), 1632 cm^−1^ (*ν*(C=C)), and 1510 cm^−1^ (aromatic skeletal vibration) were absent in the cellulose, the chemical structure of lignin a polymer found in the call walls of plants.^171^ After extraction the cellulose was carboxylated to increase its hydrophilic nature and thus increase its propensity to absorb water. This was confirmed with FTIR bands at 776, 844 and 1631 cm^−1^ as well as Raman bands at 852 cm^−1^ and 702 cm^−1^, indicative of trichloroacetic acid group. FTIR analysis also confirmed successful grafting with peaks at 1636 and 1614 cm^−1^ for *ν*(C=C), while corresponding Raman peaks at 1659 cm^−1^ and 1637 cm^−1^ seen in the monomer were absent. The 3D structure necessary for water absorption was further validated by shifts in IR band at 1690 cm^−1^ and Raman band at 1709 cm^−1^, as well as the emergence of new IR and Raman bands at 1090 and 996 cm^−1^ respectively, due to the ester formation after crosslinking. As such vibrational spectroscopy was able to provide essential information and confirmation of the synthesis of this hydrogel.^172^

In addition, a separate study utilized ATR-FTIR and XPS to characterize a hydrogel synthesized from wastewater generated after cooking rice. In this study, the functional hydrogel though it did fluoresce due to impurities in the starch during Raman measurements, it was addressed by performing a baseline subtraction. The ATR-FTIR revealed substantial peaks around 1669 cm^−1^, corresponding to the carbonyl group of the amide moiety of the acrylamide unit. Likewise, the most intense Raman peak at 2913 cm^−1^ is associated to the symmetrical and asymmetrical CH stretching, shifted to 2917 cm^−1^ due to the polymerization between starch and the AAm-*co*-AMPS polymer. XPS data further showed new peaks that confirmed that formation of a hydrogel AAm-*co*-AMPS polymer. The aim of this hydrogel synthesis was to be used in agriculture. It was found that hydrogel had a positive effect on the growth of mung beans, pea seeds, and young chili plants. The soil with hydrogels had better plant growth than control soil for all. Along with higher moisture level six days after water and biodegradation clearly show that there is a potential for using hydrogel that are environmentally friendly can be useful in everyday life.^173^

In a medically related study Raman spectra of disposable soft contact lens (SCLs) made from hydrogels were studied. Two commercially available lenses, composed of 2-hydroxyethylmethacrylate (HEMA) copolymerized with 2% methacrylic acid (MAA) were examined. These lenses are to be replaced on a daily and biweekly bases and Raman spectra of the 2 contact lenses resulted in similar spectra and proved they are both chemically bio-compatible, due to the absence of then C=C band at *ca.* 1640 cm. Also, the relative intensity of the band at 3420 cm^−1^*ν*(OH) with that at 2945 cm^−1^*ν*(CH), one can see that the two disposable lenses have the same hydrophilicity and, consequently, the same physical and biological biocompatibility. However, when placed in different pH solution the intensity of the 3420 cm^−1^ varied indicating even tiny amounts of MAA greatly affects the hydrophobicity and that pH and ionic strength of the solution both play a role. While further studies are needed to fully understand these SCLs Raman spectroscopy was critical in providing a non-destructive way of analysis as compared to other methods that have been utilized.^174^

### Electrochromic materials

These are materials that are capable of undergoing a reversible optical absorption at visible wavelength due to electrochemical stimuli, or in other words, materials that can electrochemically switch colours.^175^ Examples of materials include viologen, an organic compound that undergoes a color change upon redox reaction, and tungsten trioxide, an inorganic compound that changes color due to the dual injection of ions and electrons.

The study of electrochromic materials using Raman spectroscopy dates back several decades and covers a vast array of materials. To focus on a few is always a difficult task nonetheless here are a few examples over the decades. One compound studied over the decades is nickel oxide (NiO) due to its abundance, ease of synthesis and cost effective. As mentioned earlier, NiO exhibits a color change when subjected to a potential difference, transitioning from transparent (bleached) state to a brown (colored) state.^176,177^ This transformation is due to Ni(ii) to Ni(iii) redox in an alkaline environment. To conduct this redox reaction the sample must be placed in an electrode. In this study two deposition techniques were used to adhere the Ni oxide and investigated using UV-Vis, XPS and Raman spectroscopy. Raman spectroscopy of previous studies has reported to Ni(OH)_2_ species between 445–465 cm^−1^. However, in doped Ni(OH)_2_ a shift of 65 cm^−1^ was observed. The bleached state, at the potential of 0.2 V, contains a Raman peak at 523 cm^−1^ implying a disordered Ni(OH)_2_, a finding corroborated by XPS. Possibly sulphate ions were inserted into the Ni(OH)_2_ lattice during deposition causing Ni–O band and an additional band at 1076 cm^−1^ for sulphate ions. The colored state reveals bands at 471 cm^−1^ and 550 cm^−1^, which can be assigned to the Raman active shifts of NiOOH. XPS upon closer inspection had a significant intensity peak difference between the colored and bleached Ni samples at the 3d peak. Unlike Raman measurement, no sulphate ions were detected in the surface analysis, suggesting the sulphate ions were present in the bulk rather than the surface. This highlights the importance of taking reference Raman measurements. While high-resolution XPS was also conducted, multiplet splitting posed challenges in assigning all peaks.^176^

There are multiple studies on TMOs, such as the use of Raman to study the electrochromic effect of TiO_2_ based upon a change in temperature from 400–800 °C,^177^ while another studied single crystals of NiO in different orientations, highlighting how Raman was both complimentary and essential in understanding NiO at the molecular level.^178^

Metallic organic frameworks (MOFs) also hold promise for electrochromic applications for the mere fact of their color reversibility upon solvent exchange. By adjusting particle size and thickness, MOFs can be adjusted to match length of the decay of confined electromagnetic field in localized surface plasmons (LSPs), thereby enhancing Raman scattering. As such, MOFs have the ability to be treated like semiconductor-like light harvesters with reprogrammable optical gaps.^179^

One typical study of the electrochromic behavior of MOF was done by applying a positive electrochemical bias to Mn-MOF-74 thin films synthesized on fluorine-doped tin oxide (FTO)-coated glass. What was observed was a reversible color change from ochre to brown which was attributed to the oxidation of the 2,5-dihydroxyterephthalic acid linker (dhtp) within the MOF framework. To investigate the underlying mechanism, *in situ* Raman spectroelectrochemistry was employed. The characteristic bands for the neutral dhtp linker were identified at 560, 1284, and 1403 cm^−1^ corresponding to the C–H bending, C=O stretching and O–C–O symmetric stretching modes, respectively. During the Raman spectroelectrochemistry, these dhtp linker bands begin to diminish as the voltage was increased and a simultaneous appearance of new bands – most notably at 1625 cm^−1^ which indicated the oxidized state of the dhtp linker signifying the formation of a new C=O bond and confirming the redox activity of the dhtp linker. Theoretical modeling using DFT supported these observations, with calculated vibrational modes aligning with the experimental Raman spectra. To verify that the redox process was localized on the organic linker and not on the metal centers, the oxidation stats of the magnesium cations was assessed using X-ray photoelectron spectroscopy (XPS). The Mn 3s and 2p spectra revealed that manganese remained in the +2 oxidation state throughout the electrochemical doping process. Not only did it not end there, the Mn-MOF-74 was incorporated into an electrochromic device and it is interesting to know that the device constructed with the Mn-MOF-74 thin films exhibited reversible color changes upon alternating between positive and negative biases and also retained its electrochromic performance over 100 cycles, with only a minor reduction in charge capacity.^180^

These findings underscore the potential of Raman spectroscopy and MOF based materials in uncovering redox-drive processes and highlights their potential for real world applications.

### Thermochromic materials

These materials have a color change that is dependent on temperature. This reversible effect is a result of heating and cooling and occurs usually *via* absorption of reflection.^181^

Here, we present a few examples of thermochromic materials investigated using vibrational spectroscopy. One application of thermochromic materials is for temperature sensing. Polydiacetylenes (PDAs) are among the compounds utilized for this purpose. However, they possess one drawback: their tendency to undergo an irreversible transition. This is a result of a missing transition *via* an intermediate state when going from the ground to excited state. Despite this limitation, PDAs are of great interest because unlike other conjugated polymers, PDAs are functionalized *via* photopolymerization of the self-assembled monomers.^182^ An extensive look into differently conjugated thermochromic polymers assembled *via* Langmuir-Blodgett (LB)/Langmuir–Schaefer (LS) and analysis using FTIR that laid a foundation for future studies can be found here.^182^ FTIR established that it a crucial factor for reversibility is to have strong hydrogen-bonding and aromatic interactions which are retained at high temperature. These interactions enable regeneration of the conjugated π-electron system upon stimulus removal.

To summarize some of the findings experiments that to a color change due to thermal stimuli and hydrogen-bonding interactions strongly propose that cooperative and integrated interactions between amide, aromatic, and carboxylic acid head-groups are a necessary condition for achieving color reversibility of PDAs.^182^

**Fig. 11:**
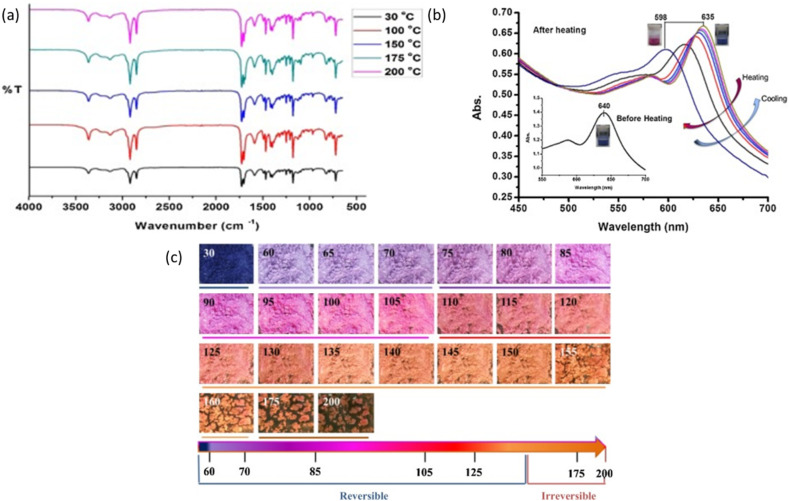
(a) ATR-FTIR spectra of poly-pcd-urea following thermal cycling at various temperatures (b) UV-Vis spectra illustrating reversible color changes in poly-pcd-urea sols during heating (RT to ∼100 °C) and subsequent cooling. The spectra were recorded from the red phase as the sols were cooling down to the blue phase. (c) Photographs of poly-pcd-urea powders after equilibration at 30–200 °C, with a schematic rainbow bar indicating color transition trends (temperature graduation: not to scale). All figures are reproduced with permission.

A similar more recent study found that by adding urea to 10,12-pentacosadiynoic acid (PCDA) a type of PDA aided in overcoming this limitation due to having a high number of hydrogen bonding sites.^183^ FTIR was used to corroborate the NMR results as it clearly identified urea peaks embedded in the polymer, while ATR-FTIR and UV/VIS monitored the changes seen with temperature increase. It was found that up to 200 °C FTIR remained unchanged and attributed this to strong electrostatic interactions in the head groups shielding the pi-conjugated backbone (Fig. 11a).^183,184^ Furthermore, UV/Vis showed the transition of the poly-pcd-urea compound return to its original state after heating and cooling. The recorded UV had an absorption at 640 nm and blue color prior to heating at 100 °C. At 100 °C a red color was seen and the wavelength shifted to 598 nm and upon cooling back to room temperature, it returned to 635 nm and its blue color (Fig. 11b and c). With these findings it was clear that urea's incorporation was beneficial and as such highlighted the potential for this PCDA to be used in visual temperature sensing.^183^ Further and more in-depth reading of thermochromic materials can be found here.^181,185,186^

## Conclusions and perspective

Functional and smart materials are redefining the landscape of modern material science through exhibiting predictable changes in properties or behavior such as shape, color, stiffness, or thermal conductivity in response to external stimuli (*e.g.*, stress, temperature, moisture, change in pH-value, electric, or magnetic fields). The change of the properties is often caused by structural changes within the materials. Consequently, precise knowledge about the ongoing molecular changes is indispensable, since minute changes in the molecular structure can already result in drastic changes of the material's properties.

In this context, this review has highlighted how label-free linear and non-linear vibrational spectroscopy techniques, such as IR and Raman spectroscopy, are instrumental for understanding the molecular structures and, in particular, also their respective changes and dynamic behavior upon application of the stimulus.

The advantage of the present techniques is that they not only provide non-invasive, high-resolution, and real-time insights but also enable researchers to explore dynamic behaviors and establish correlations between molecular structures and their macroscopic (switchable) properties. As functional materials continue to advance, the integration of these advanced spectroscopic techniques will continue to play a critical role in driving innovation and broadening their application across a variety of fields. Beyond current capabilities, ongoing efforts in the development of the next generation spectroscopic methods are expanding the boundaries of what can be measured and understood. This advancement requires pushing the limits of spatial and temporal resolution, sensitivity and selectivity in spectroscopic techniques. One innovative approach is the superresolution single-band coherent Raman scattering (CRS) imaging. In this method, two synchronized laser pulses, a picosecond pump and a femtosecond probe, interact to generate vibrational coherences within the sample. This is followed by a spatially donut-shaped decoherence beam to selectively eliminate the vibrational coherence in the outer ring of the focal region. This means that only the central region retains coherence, allowing stimulated Raman signal generation to occur exclusively within this sub-diffraction-limited area, thereby improving spatial resolution.^187^ The principle behind this technique is inspired by the Stimulated Emission Depletion (STED) fluorescence microscopy, where a similar donut-shaped decoherence beam suppresses fluorescence around the edges, confining emission to the center and thus achieving superresolution.^188^

Another innovative approach is the broadband CARS, which facilitates the rapid acquisition of full Raman spectra across a broad range (400–3200 cm^−1^) within milliseconds.^72^ Here, two laser pulses, pump beam and stoke beam, interact with the sample to excite molecular vibrations and thus generate a coherent signal. But instead of probing one vibrational mode at a time, broadband CARS uses a broadband Stokes beam to excite and detect many vibrations simultaneously. This capability allows for fast, label-free chemical imaging of complex samples, providing comprehensive spectral information in a single measurement.

It is also interesting to see the integration of Raman spectroscopy with other modalities like Tip-Enhanced Raman spectroscopy coupled with Atomic Force Microscopy (TERS-AFM)^189^ as being part of the forefront of these innovations. This enables simultaneous topographical mapping and molecular characterization at the nanoscale, with TERS demonstrations approaching angstrom-scale resolution under optimized conditions.^74,190^ Ongoing work is also focused on translating such performance to ambient and *operando* environments while maintaining quantitative reliability. Advances in tip engineering, plasmonic stability, and drift/feedback control are expected to narrow the gap between best case demonstrations and day to day practice.

In parallel, nano-IR techniques, including AFM-IR and PiFM, are rapidly advancing in sensitivity, speed, and spectral resolution. Particular attention is turning to sample-geometry considerations such as ultrathin films, rough or porous surfaces, multilayers, and buried interfaces which strongly influences local field enhancements, thermal diffusion volumes and photothermal/mechanical transduction. Incorporating geometry-aware forward models, calibrated reference standards, and robust uncertainty estimates will make nano-IR measurements more quantitative and transferable across materials.^54,191^

Concurrently, AI and ML are being integrated across full vibrational analysis pipeline. That is, from baselining, spike removal, and hyperspectral unmixing/segmentation to automated band assignments, uncertainty-aware peak fitting, inverse modelling of structure to property relationship. When paired with standardized metadata and open datasets, these methods can convert large spectroscopic data into reproducible, quantitative molecular maps and accelerate discovery in functional materials.^82–84^

A further frontier is emerging where 3D/volumetric vibrational imaging and correlative multimodal workflows. Depth-resolved AFM-IR, OPTIR tomography and volumetric coherent Raman imaging are beginning to reveal buried architectures and interphases relevant to device operation. When paired with complementary probes, *e.g.*, electron microscopy, X-ray nanoprobes, mechanical mapping, these approaches provide multiscale, correlative views that connect chemistry, morphology, and function.^52,192,193^

Collectively, these advanced spectroscopic techniques and many others are redefining the limits of spatial resolution, sensitivity and selectivity and serve as powerful tools to explore the dynamic molecular intricacies of various material systems.

## Author contributions

Writing of manuscript: M. F. A., A. L. J. L. E., M. D. H., M. S..; supervision: M. S., J. P.; correction of the manuscript: M. F. A., A. L. J. L. E., S. Z., M. D. H., M. S., J. P.

## Conflicts of interest

The authors state that they have no known financial or personal conflicts of interest that could have influenced the work reported in this paper.

## Supplementary Material

SC-016-D5SC04114G-s001

## Data Availability

This article is a review and does not include any original data. All data discussed are derived from previously published studies, which are appropriately cited in the text and reference list. No new data were generated or analyzed in support of this work. Permissions to reproduce figures from previously published work have been obtained and are included in the supplementary information (SI). Supplementary information is available. See DOI: https://doi.org/10.1039/d5sc04114g.
