# Supplementary material for: Label-free linear and non-linear vibrational spectroscopy for functional materials: state-of-the-art and future perspectives
Source: Chem Sci. 2025 Nov 4;16(48):22826–51. doi: 10.1039/d5sc04114g (PMC12612856; doi:10.1039/d5sc04114g)
Supplement: SC-016-D5SC04114G-s001 [file SC-016-D5SC04114G-s001.pdf]

Supplementary Information (SI) for Chemical Science.
This journal is © The Royal Society of Chemistry 2025

ARTICLE

Label-free linear and non-linear vibrational spectroscopy for functional materials: state-of-the-art and future perspectives

Received 00th January 20xx,
Accepted 00th January 20xx

DOI: 10.1039/x0xx00000x

Michael Freduah Agyemang*[a], Akuila Edwards[a], Stefan Zechel[c,d], Martin D. Hager[c,d,e,f], Michael Schmitt[a], Juergen Popp[a,b]

[a] *Institute of Physical Chemistry (IPC), Abbe Center of Photonics (ACP), Friedrich Schiller University Jena, Helmholzweg 4, 07743 Jena, Germany.*
[b] *Leibniz Institute of Photonic Technology, e.V. Jena, Albert-Einstein-Str. 9, 07745 Jena, Germany.*
[c] *Laboratory of Organic and Macromolecular Chemistry (IOMC), Friedrich Schiller University Jena, Humboldstr. 10, 07743 Jena, Germany.*
[d] *Jena Center of Soft Matter (JCSM), Friedrich Schiller University Jena, Philosophenweg 7, 07743 Jena, Germany.*
[e] *HIPOLE Jena, Philosphenweg 12-14, 07743 Jena, Germany*
[f] *Helmholtz Zentrum Berlin für Materialien und Energie Gmb*

† Footnotes relating to the title and/or authors should appear here.
Supplementary Information available: [details of any supplementary information available should be included here]. See DOI: 10.1039/x0xx00000x

Table 1: A comprehensive comparison of Raman and IR spectroscopy techniques[1-3].

	Raman spectroscopy	Infrared spectroscopy
Principle	Measures the inelastic scattering (Stokes and anti-Stokes) of EM radiation	Measures the absorption transitions between vibrational levels in the electronic ground state.
Selection rules	Vibration is Raman active if it causes a change in polarizability of the molecule	Vibration is IR active if there is a change in dipole moment of the molecule
	In centrosymmetric molecules, modes that are Raman-active are IR-inactive and vice versa (mutual exclusion rule)	
Instrumentation	Uses a monochromatic laser source, beam splitters, and detectors such as charge-coupled devices (CCDs)	Uses a broadband infrared light source, monochromators or interferometers, and detectors like pyroelectric devices
Sensitivity	Sensitive to non-polar bonds involving atoms of similar electronegativity, such as C=C and C≡C bonds	Highly sensitive to polar bonds and functional groups with significant dipole moments, such as O-H, N-H, and C=O bonds
Sample preparation and compatibility	Water has a weak Raman scattering effect, allowing for the analysis of aqueous solutions<br>Suitable for samples in various states<br>Minimal sample preparation is needed	Water can strongly absorb IR radiation, making it challenging to analyze aqueous solutions<br>Sample thickness and concentration need careful control to avoid saturation<br>Require specific sample preparation
Interference factors	Under visible excitation, fluorescence chromophores or trace impurities in the sample itself can overshadow Raman signals and therefore a careful selection of excitation wavelength and sample purity is important	Strong IR absorption by water, CO_2, pathlength saturation in transmission, and scattering/dispersion in rough or filled samples can interfere with measurements.<br>Sample preparation and environmental conditions need careful control
Sampling mode	Includes 180° backscattering modes, confocal and transmission and 90°.	Geometries include transmission, attenuated total reflection (ATR), or Diffuse Reflectance Infrared Fourier Transform (DRIFT), and specular reflection

Please do not adjust margins

1. J. R. Ferraro, K. Nakamoto, C. W. Brown and ScienceDirect, *Introductory Raman spectroscopy*, Academic Press, Amsterdam ; Boston, 2nd edn., 2003.
2. G. Socrates, *Infrared and Raman characteristic group frequencies : tables and charts*, Wiley, Chichester, 3rd edn., 2001.
3. R. R. Jones, D. C. Hooper, L. W. Zhang, D. Wolverson and V. K. Valev, Raman Techniques: Fundamentals and Frontiers, *Nanoscale Res Lett*, 2019, **14**.

Please do not adjust margins
